# Carbazole scaffolds in cancer therapy: a review from 2012 to 2018 

**DOI:** 10.1080/14756366.2019.1640692

**Published:** 2019-07-22

**Authors:** Samar Issa, Anthony Prandina, Nicolas Bedel, Pål Rongved, Saïd Yous, Marc Le Borgne, Zouhair Bouaziz

**Affiliations:** a Ecole de Biologie Industrielle, EBInnov, Cergy-Pontoise, France;; b Faculté de Pharmacie - ISPB, EA 4446 Bioactive Molecules and Medicinal Chemistry, SFR Santé Lyon-Est CNRS UMS3453 - INSERM US7, Université de Lyon, Université Claude Bernard Lyon 1, Lyon, France;; c Department of Pharmaceutical Chemistry, School of Pharmacy, University of Oslo, Oslo, Norway;; d Université Lille, Inserm, CHU Lille, UMR-S 1172 JPArc Centre de Recherche Jean-Pierre Aubert Neurosciences et Cancer, Lille, France

**Keywords:** Carbazole, cancer, cytotoxicity, targeted therapy, enzyme inhibitors

## Abstract

For over half a century, the carbazole skeleton has been the key structural motif of many biologically active compounds including natural and synthetic products. Carbazoles have taken an important part in all the existing anti-cancer drugs because of their discovery from a large variety of organisms, including bacteria, fungi, plants, and animals. In this article, we specifically explored the literature from 2012 to 2018 on the anti-tumour activities reported to carbazole derivatives and we have critically collected the most significant data. The most described carbazole anti-tumour agents were classified according to their structure, starting from the tricyclic–carbazole motif to fused tetra-, penta-, hexa- and heptacyclic carbazoles. To date, three derivatives are available on the market and approved in cancer therapy.

## 1. Introduction

**Figure 1.**
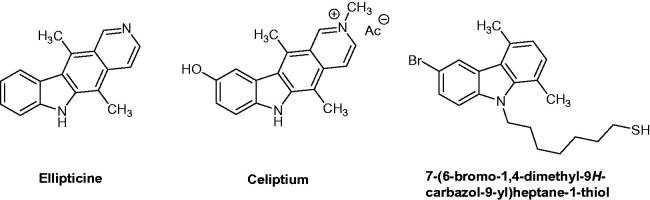
Chemical structures of ellipticine, elliptinium acetate (Celiptium^®^) and a 6-bromo derivative of carbazole.

Cancer is characterized by an uncontrolled growth of cells, which can spread to distant sites of the body with severe health consequences and is the second leading cause of death worldwide^1^. Around 14.1 million new cancer cases and 8.2 million cancer-related deaths occurred in 2012, and 29.4 million new cases are estimated for 2035 with 18.8 million cancer-related deaths (GLOBOCAN 2012)^2^. The most commonly diagnosed cancers worldwide are those of the lung (1.8 million, 12.6% of the total), breast (1.7 million, 11.9%), colorectal (1.4 million, 9.8%) and prostate (1.1 million, 7.7%) cancers. Keeping in mind that both cancer cases are increasing and resistance to anti-cancer drug regimens are emerging, research and development of new powerful cancer treatments became extremely crucial for the next decades. Among the existing anti-cancer drugs, the carbazole scaffolds have been, for over half a century, the key structural motif of many biologically active compounds including natural and synthetic products^3^. Carbazole alkaloids originate in most cases from higher plants of the genera *Murraya*, *Glycosmis*, *Clausena* and *Micromelum*, all from the family of Rutaceae^4^. Other sources are bacteria (e.g. *Streptomyces*), algae (e.g. *Hyella caespitosa*) and fungi (e.g. *Aspergillus* species). The parent compound *9H*-carbazole was isolated from coal tar in 1872 by Graebe and Glazer^5^. The first naturally occurring carbazole, the alkaloid murrayanine, was isolated from *Murraya koenigii* Spreng in 1962^6^. Later, many carbazole derivatives have been synthesized and are well known for their pharmacological activities such as anti-oxidant, anti-inflammatory, anti-bacterial, anti-tumour, anti-convulsant, anti-psychotic and anti-diabetic^7^. Many carbazole derivatives and related compounds have been studied. More interestingly, three derivatives have obtained marketing authorization with anti-cancer drug status in different countries. Ellipticine, which was discovered in 1959 (Figure 1) and extracted from the leaves of *Ochrosia elliptica* (Apocynacae) before being entirely synthesized, could be considered as the first initial lead compound of carbazole analogues. Thereafter, an ellipticine analogue named *N*-methyl-*9*-hydroxyellipticinium acetate (Celiptium^®^) has been developed. Since 1982, Celiptium^®^ is still currently used in the treatment of metastatic breast cancer^8^^,^^9^. As reported by the National Cancer Institute (NCI) drug dictionary^10^, *N*-methyl-*9*-hydroxyellipticinium acetate acted as a topoisomerase II inhibitor and an intercalating agent, stabilizing the cleavable complex of topoisomerase II and inducing DNA breakages, thereby inhibiting DNA replication and RNA and protein synthesis. New *N*-thioalkylcarbazole derivatives were synthesized and evaluated in comparison to ellipticine. Among the bioactive carbazole-type derivatives, 7-(6-bromo-1,4-dimethyl-9*H*-carbazol-9-yl)-heptane-1-thiol (Figure 1) needs to be also mentioned^9^.

**Figure 2.**
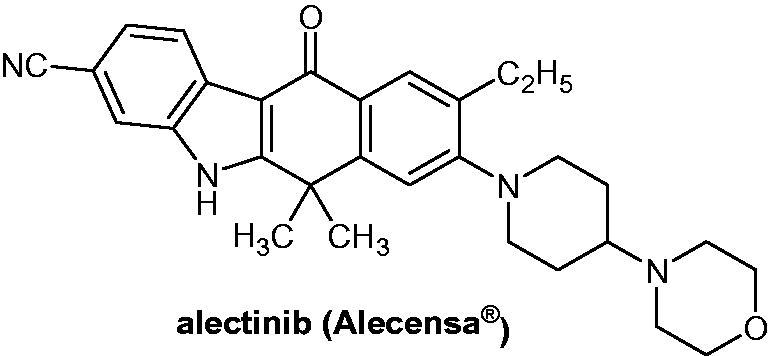
Chemical structure of alectinib, an ALK inhibitor.

The second derivative to obtain marketing authorization was alectinib bearing a *5H*-benzo[b]carbazol-11(6*H*)-one scaffold (AF802, CH 5424802, RG7853, RO5424802, Alecensa^®^) (Figure 2). Alectinib, an orally available drug, was first approved in 2015 by the US Food and Drug Administration (FDA)^11^ for Genentech and then by the European Medicines Agency (EMA) for Roche Pharmaceuticals^12^, with an indication as monotherapy for the treatment of adult patients with anaplastic lymphoma kinase (ALK)-positive advanced non-small-cell lung cancer (NSCLC)^13^.

**Figure 3.**
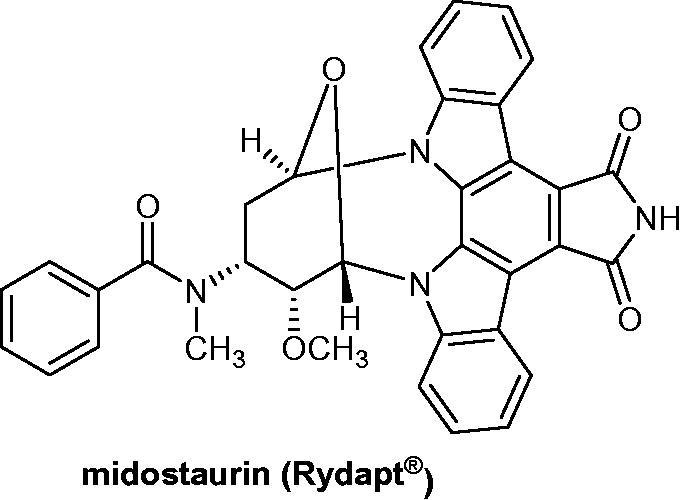
Chemical structure of midostaurin, an FLT3 inhibitor.

The third derivative recently approved in 2017 by the FDA^14^ and the EMA^15^ is midostaurin (CGP41251, PKC412, Rydapt^®^) (Novartis) (Figure 3), described mainly as the first fms-like tyrosine kinase 3 (FLT3) inhibitor for newly diagnosed acute myeloid leukemia (AML) and for advanced systemic mastocytosis (SM)^16^^,^^17^.

**Figure 4.**
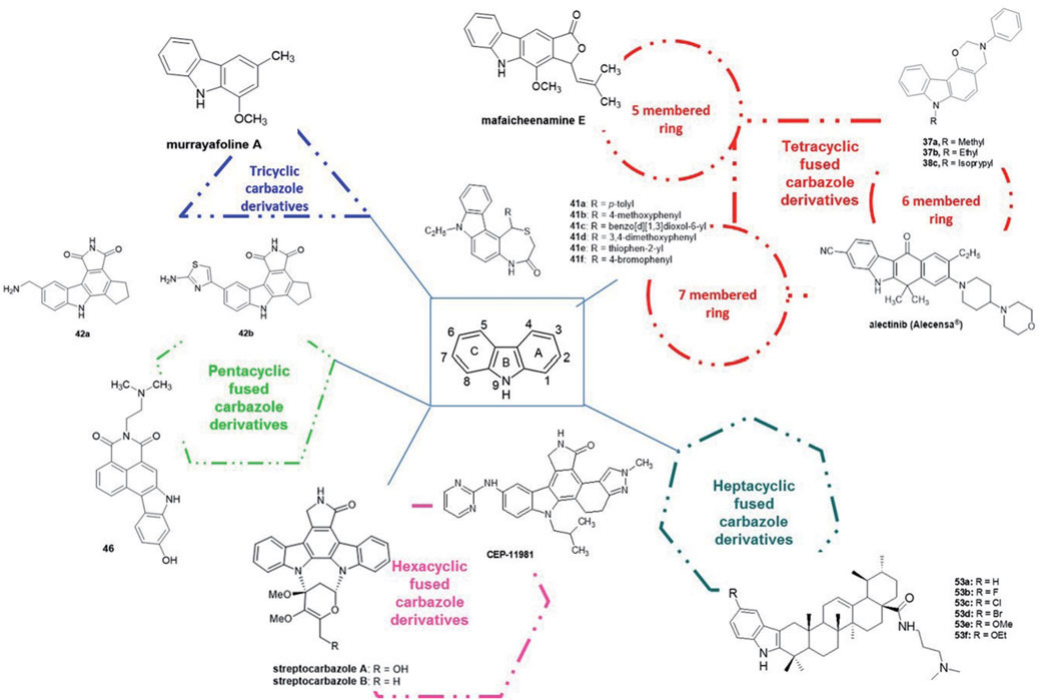
Main frameworks of biologically active carbazole alkaloids.

Compared to the previously recent published reviews^18^, we focused this article on the carbazole derivatives exerting anti-tumour activity reported from 2012 to 2018, and we critically collected the most significant data. The term “carbazole” includes both the tricyclic molecular skeleton and diverse fused carbazoles including tetracyclic (with 5-, 6- and 7-membered rings), pentacyclic, hexacyclic and finally heptacyclic fused carbazoles (Figure 4). Several databases, bibliographic information (articles) from namely ScienceDirect, Scifinder, Pubmed and Web of Science as well as technological (patents) information from INPI Patents Database, European Patent Office (EPO), as well as the World Intellectual Property Organization (WIPO), were used as literature sources.

The increased interest in the use of carbazole derivatives for the cancer therapy can also be expressed in the research of patents. In Table 1, the publication of 10 patents^19–28^ is summarized including one European patent, three US patents and seven international patents for carbazole derivatives currently described for their anti-cancer activity.

**Table 1.** Overview of the major patents since 2012 related to carbazole derivatives as anti-cancer agents.

Patent number	Year of publication	Inventors	Title	Structure
EP2403855 B1	2013	Ahmed et al.	Hybrids of carbazole-bound pyrrolo [2,1-*c*][1,4]benzodiazepine as potential anti-cancer agents and their method of preparation^19^	
US8815840 B2	2014	Purandare et al.	Carbazole and carboline kinase inhibitors^20^	
WO2012059232 A1	2012	Demotz S et al.	Carbazole and carboline derivatives, their preparation and therapeutic applications thereof^21^	
US20120184590 A1	2012	Rawjewski et al.	Formulations of indole-3-carbinol derived anti-tumour agents with increased oral bioavailability^22^	
WO2013121385 A1	2013	Rault et al.	Use of carbazole-phenone derivatives for treating cancer^23^	
WO2014134232 A1	2014	Poss et al.	Carbazole compounds useful as bromodomain inhibitors^24^	
US20160024083 A1	2016	Gurova et al.	Compounds and methods for treating cancers^25^	
US20140303224 A1	2014	Tucker et al.	Carbazole compounds and therapeutic uses of the compounds^26^	
US2017158636 A1	2017	James et al.	Functionalized and substituted carbazoles as anti-cancer agents^27^	
US2017166526 A1	2017	Narayanan et al.	Selective androgen receptor degrader (SARD) ligands and methods of use thereof^28^	

## 2. Tricyclic carbazoles

### 2.1. Ferrocenyl platinum(II) complex

**Figure 5.**
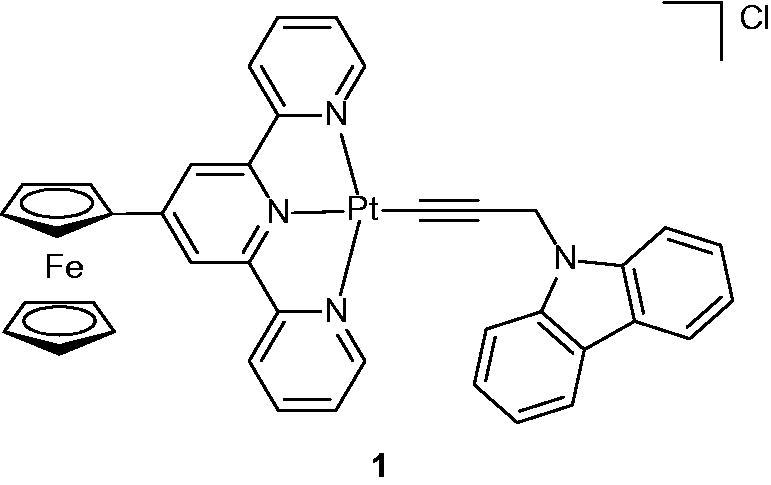
Chemical structure of a ferrocenyl–terpyridine platinum(II) complex **1**.

Ferrocenyl platinum(II) complex [Pt(Fc-tpy)(NPC)]Cl (HNPC = N-propargylcarbazole) was synthesized and evaluated for its anti-proliferative properties in visible light against HaCaT (human keratinocyte) cell lines. Compound **1** (Figure 5) exhibited interesting photocytotoxicity in HaCaT cell lines with an IC_50_ value of 12.0 µM in visible light (400–700 nm) with low dark toxicity (IC_50_>60 µM)^29^.

### 2.2. N-Acylcarbazoles

**Figure 6.**
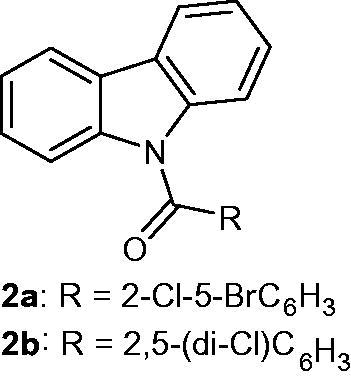
Chemical structures of *N*-acyl carbazole derivatives **2a** and **2b**.

*N*-Acylated carbazoles were synthesized and evaluated for their anti-proliferative activities against CAL 27 (squamous cell carcinoma) cell lines. The IC_50_ values of the most active compounds **2a** and **2b** (Figure 6) were 0.028 and 0.45 µM, respectively^30^.

### 2.3. 6-Methyl-2,3,4,9-tetrahydro-1H-carbazoles

**Figure 7.**
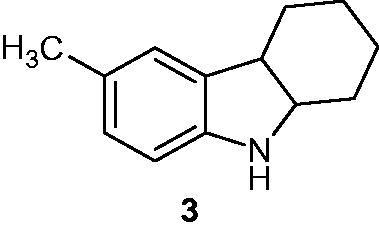
Chemical structure of tetrahydrocarbazole **3**.

Tetrahydrocarbazoles were synthesized and tested for anti-cancer activity against six different cell lines, namely human kidney adenocarcinoma (ACHN), pancreas carcinoma (Panc1), lung carcinoma (GIII and Calu1), non-small-cell lung carcinoma (H460), human colon carcinoma (HCT116) and normal breast epithelium (MCF10A) cell lines. Carbazole derivatives demonstrated moderate to good activities and among them, compound **3** (Figure 7) was found to be the most active against Calu1 cell line with an IC_50_ of 2.5 nM^31^.

### 2.4. N-{3-[3–(9-Methyl-9H-carbazol-3-yl)-acryloyl]-phenyl}-benzamide

**Figure 8.**
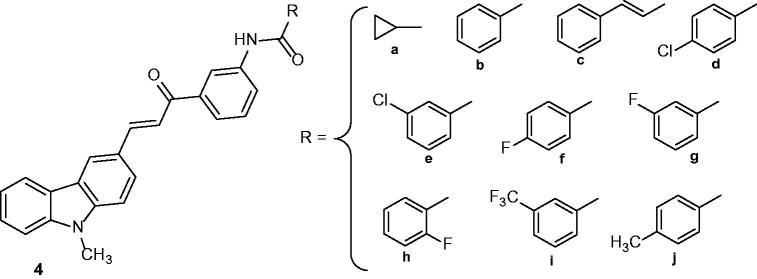
Chemical structures of N-{3-[3–(9-methyl-9*H*-carbazol-3-yl)-acryloyl]-phenyl}-benzamide derivatives **4a–j**.

These derivatives (Figure 8) were synthesized and evaluated for their in vitro xanthine oxidase (XO), tyrosinase and melanin production inhibitory activity. Most of the target compounds (**4a**, **4c**, **4d**, **4e**, **4g**, **4i** and **4j**) inhibited XO with IC_50_ values comprised between 4.3 and 5.6 µM. Furthermore, these derivatives showed a better activity than the standard drug allopurinol (IC_50_ value of 8.5 µM). Interestingly, compound **4a** bearing a cyclopropyl ring was found to be the most potent inhibitor of XO with an IC_50_ of 4.3 µM. Compounds **4b**, **4d**, **4f**, **4h** and **4j** were found to be potent inhibitors of tyrosinase (IC_50_ values ranging from 14.01 to17.52 µM). These results suggest the possible use of these compounds for the design and development of novel XO and tyrosinase inhibitors^32^.

### 2.5. 3,6-Bis(1-methyl-4-vinylpyridinium) carbazole diiodide (BMVC)

**Figure 9.**
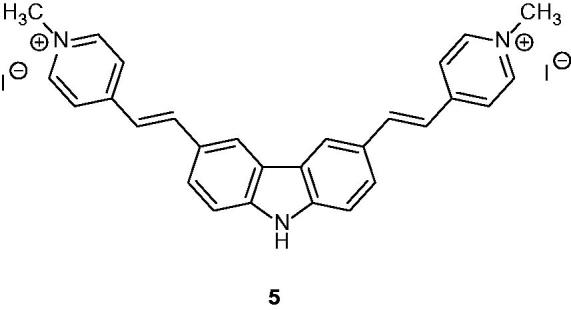
Chemical structure of BMVC or compound **5**.

BMVC or compound **5** (Figure 9) is known for its ability (i) to suppress the telomerase activity, (ii) to induce senescence of cancer cells and (iii) to destroy the intra-tumour vasculature. BMVC was studied for tumour targeting as well as for its photo-induced anti-tumour effect. The properties of this fluorescent molecule provided a design of photosensitizer (PS) for photodynamic therapy (PDT) treatment. PDT results showed that BMVC inhibited the growth of tumour cells both in vitro and in vivo^33^. BMVC is the most studied to this date as “G-quadruplex” ligand, which interacted with different forms of nucleic acids and stabilizes G-quadruplex structures. BMVC suppressed the tumour-related properties of cancer cells, including cell migration, colony-forming ability and anchorage-independent growth^34^. In clinical tests (overall, 114 outpatients), the use of fluorescent BVMC was investigated for the cancer diagnosis (needle aspirates of neck masses)^35^. Many analogue derivatives of BVMC have been analysed and used as probes due to the fluorescent electron donating optical chromophore properties of this carbazole derivative. Recently a new derivative has been used for the detection of bcl-2 2345 quadruplex structures^36^.

### 2.6. Benzopsoralen and 3-hydroxy-N-alkylcarbazole

**Figure 10.**
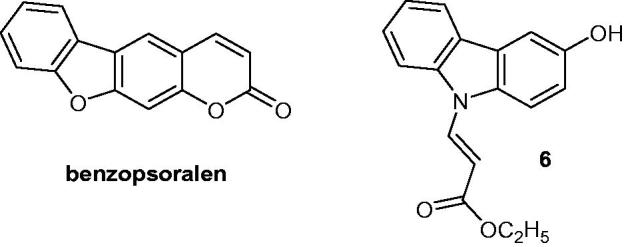
Chemical structures of benzopsoralen and (*E*)-ethyl-3-(3-hydroxy-9*H*-carbazol-9-yl)acrylate **6**.

Benzopsoralen derivatives and its carbazole analogues were synthesized, tested against MDA MB231 (breast carcinoma) and TCC-SUP (urinary bladder cell carcinoma) cell lines, and their mechanism of action was investigated by means of molecular docking studies. Every benzosporalen and carbazole derivative showed interesting anti-proliferative activities, with GI_50_ values in the nanomolar range against both cell lines. Among carbazole derivatives, compound **6** (Figure 10) had very strong activity with GI_50_ values of 0.198 and 0.025 µM against MDA MB231 and TCC-SUP cell lines, respectively^37^.

### 2.7. MHY407

**Figure 11.**
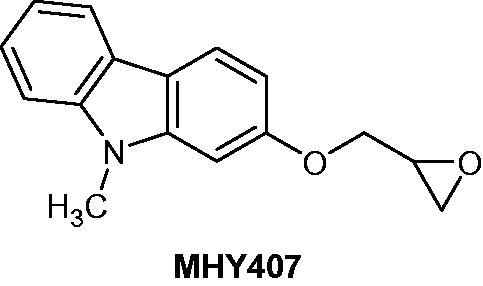
Chemical structure of MHY407.

The carbazole derivative **MHY407** (Figure 11) is active against breast cancer cell lines by inhibiting cellular proliferation with IC_50_ around 5 µM. This compound increased DNA damage and triggered cell cycle arrest in S phase. In combination with various chemotherapeutic treatments, such as doxorubicin, etoposide or radiation, **MHY407** improved the efficiency of the treatments by reducing cell viability and increasing apoptosis^38^.

### 2.8. Amide-containing carbazole derivatives

**Figure 12.**
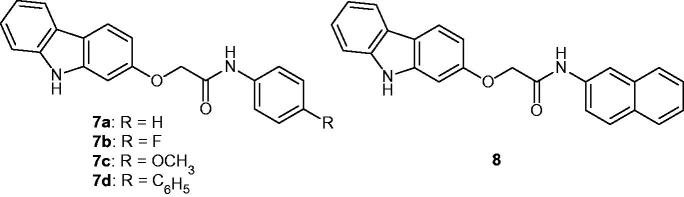
Chemical structures of amide containing carbazole derivatives **7a–d** and **8**.

Amide-containing carbazole derivatives **7a–d** and **8** (Figure 12) have been synthesized and their in vitro anti-proliferative activities against NPC-TW01 (nasopharyngeal carcinoma), NCI-H661 (lung carcinoma) and Jurkat (leukaemia) cell lines were evaluated. All carbazole derivatives were inactive or weakly active, with IC_50_ values ranging from 11.09 to 42.77 µM^39^.

### 2.9. Murrayafoline-A

**Figure 13.**
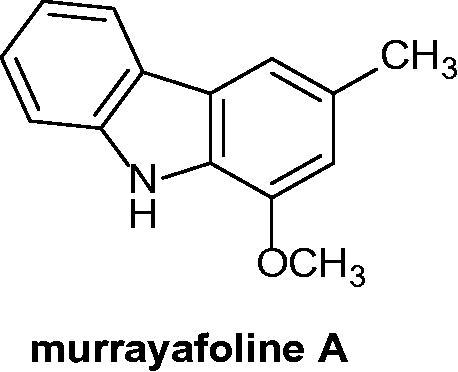
Chemical structure of murrayafoline A.

Isolated from *Murraya euchrestifolia* (Rutaceae), 13 carbazole alkaloids were evaluated against HL-60 leukaemia cell line. Murrayafoline A (Figure 13) displayed a significant interaction with the caspase-9/caspase-3 pathway, leading to the cellular apoptosis^40^.

### 2.10. Clauszoline-I

**Figure 14.**
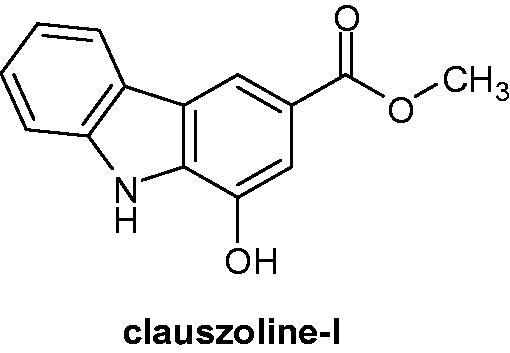
Chemical structure of clauszoline-I.

Extracted from *Clausena vestita Tao,* clauszoline-I (Figure 14) showed effective ability to induce the cell cycle arrest in the S and G2/M phases. The mechanism is linked with the inhibition of the phosphorylation of the Ser-643 of the protein kinase C delta (PKCδ). PKCδ is a prototypical class of serine/threonine kinases, and implicated in nearly all stages of cancer^41^, and the induction of the cell cycle arrest. Clauszoline-I displayed a growth inhibitory activity against four cancer cell lines (cervical carcinoma, glioblastoma, nasopharyngeal carcinoma, hormone-independent breast cancer), with IC_50_ values in the micromolar range (13.3–71.6 µM)^42^.

### 2.11. 6-Methoxy-1,4-dimethyl-N-alkylcarbazole derivatives

**Figure 15.**
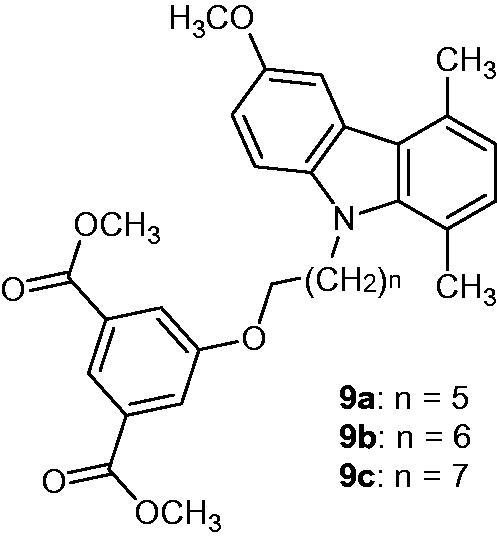
Chemical structures of *N*-alkylcarbazole derivatives **9a–c**.

Synthesized *N*-alkylcarbazole derivatives **9a–c** (Figure 15) showed interesting anti-proliferative activities. Their selective properties allowed the suppression of STAT3 phosphorylation, which led to the decrease of its mediated transcription with inhibition of 50, 90 and 95%, respectively^43^.

### 2.12. Excavatine A

**Figure 16.**
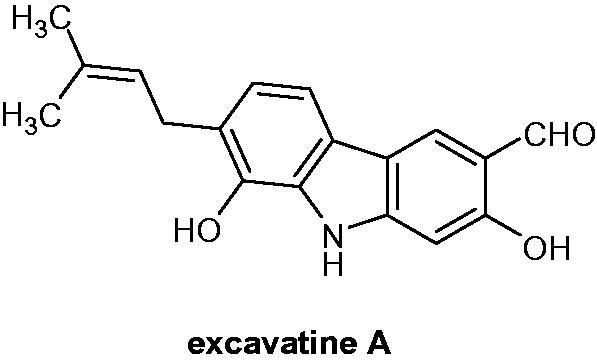
Chemical structure of excavatine A.

A carbazole alkaloid, excavatine A (Figure 16), was isolated from the stems and leaves of *Clausena excavata* BURM. f. (Rutaceae) and its cytotoxic activities against A549 lung carcinoma and HeLa cervix adenocarcinoma cell lines were assessed, showing IC_50_ values of 17.77 and 6.47 µM, respectively^44^.

### 2.13. Clausenawalline F

**Figure 17.**
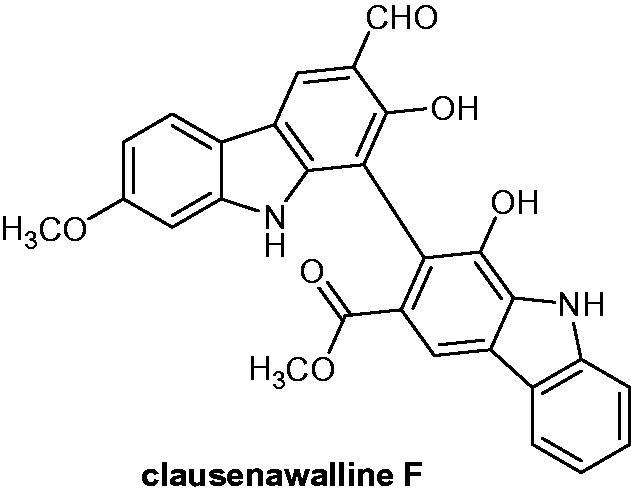
Chemical structure of clausenawalline F.

Twenty-two compounds were isolated from the roots of *Clausena wallichii* (Rutaceae) and tested for both anti-bacterial and cytotoxicity activities. After evaluations, clausenawalline F (Figure 17) exhibited the highest cytotoxicity with IC_50_ values of 10.2 µM against KB, a subline of the ubiquitous keratin-forming tumour cell line, and of 4.5 µM against NCI-H187 small-cell lung cancer cell line^45^.

### 2.14. 2-[(9-Ethyl-9H-carbazol-3-yl)amino]-2-oxoethyl N,N-disubstituted dithiocarbamate derivatives

**Figure 18.**
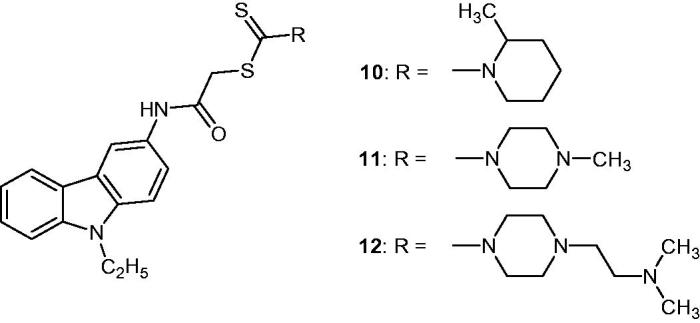
Chemical structures of compounds **10**, **11** and **12**.

Carbazole derivatives bearing dithiocarbamate group showed cytotoxic activity on C6 glioma and A549 lung carcinoma cell lines: analysis of DNA synthesis and detection of apoptosis by flow cytometry were set up. Çiftiçi et al. reported that compound **12** (Figure 18) was the most active against C6 cell line (IC_50_ value of 12.2 µM) but showed less activity against A549 (IC_50_ value of 84.7 µM). Compounds **10** and **11** (Figure 18) showed interesting activity against C6 cell line, with IC_50_ values of 62.7 and 49.9 µM, respectively^7^^,^^46^.

### 2.15. EHop-016

**Figure 19.**
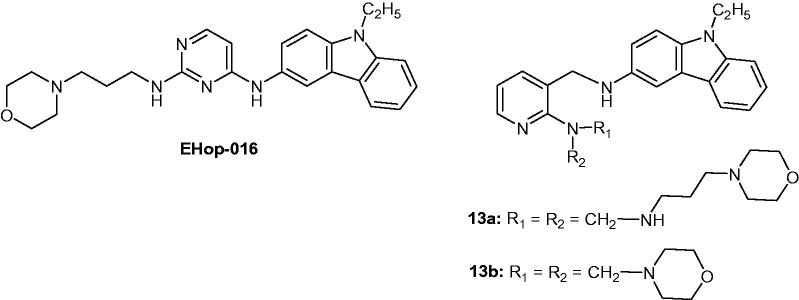
Chemical structures of EHop-016 and compounds **13a** and **13b**.

EHop-016 was serendipitously discovered to be the most potent Rac1 inhibitor. The small GTPase Rac1 is a member of the Ras superfamily of GTPases and has been implicated in the regulation of cellular migration and invasion in breast cancer cells. EHop-016 reduced metastatic cancer cell viability at a concentration inferior to 5 µM. Additionally, its anti-cancer activity (tumour growth and metastasis) was demonstrated in vivo by using a mouse model of breast cancer. The carbazole group contributed to Rac1 inhibitory activity and then new compounds **13a** and **13b** (Figure 19) were designed and synthesized. The most potent Rac1 inhibitor was **13b**, which inhibited by 55% at a concentration of 250 nM and was four times more potent inhibitor of Rac1 than EHop-016 with reduced cellular toxicity^47^.

### 2.16. Carbazole-3,6-diamine derivatives

**Figure 20.**
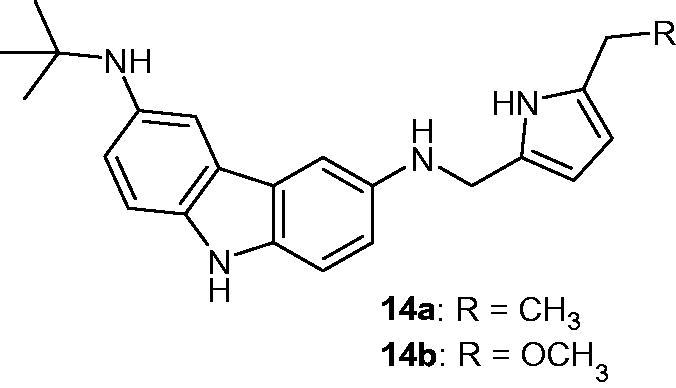
Chemical structures of carbazole-3,6-diamine derivatives **14a** and **14b**.

Carbazole derivatives bearing diamine groups presented a new potential for telomerase inhibition. Using three different docking programs (CDOCKER, Ligandfit, Autodock) and interaction analysis demonstrated that compounds **14a** and **14b** (Figure 20) had the best telomerase inhibition activity more interestingly with the introduction of a pyrazole ring^48^.

### 2.17. Carbazole sulfonamide derivatives

**Figure 21.**
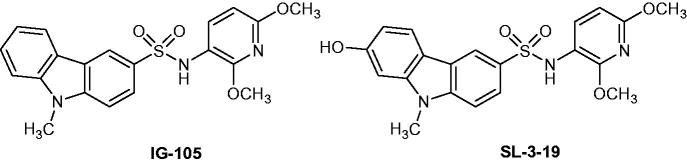
Chemical structures of compounds IG-105 and SL-3-19.

The carbazole sulfonamide IG-105^49^ (Figure 21) was described as a potent anti-mitotic agent that inhibited microtubule assembly through specific interactions within the tubulin structure. The introduction of a hydroxyl group (7-OH) on the carbazole-ring increased the solubility and provided a new derivative named SL-3–19 (Figure 21). This compound was active against HepG2 liver cancer (IC_50_ = 12 nM) and MCF-7 (IC_50_ = 14 nM) breast cancer cell lines. The IC_50_ of the positive controls podophyllotoxin and combretastatin CA-4^49^ against HepG2 were 3 and 2 nM, respectively, and against MCF-7 (IC_50_ = 20 and 5 nM, respectively). Niu et al. described further investigations on the anti-oesophageal squamous cell carcinoma (ESCC) activity and mechanisms of SL-3–19 in vitro and in vivo. Mechanistically, SL-3–19 inhibited ESCC cell growth by inducing cell apoptosis and arresting the cell cycle at G2/M phase in a dose-dependent manner. In addition to microtubule assembly inhibition, this compound showed a significant disruption of the vascular structure by obstructing the formation of capillary-like tubes in vitro and the growth of ESCC xenografts and microvessel density in vivo^50^.

### 2.18. Trimethoxybenzamide and trimethoxyphenylurea

**Figure 22.**
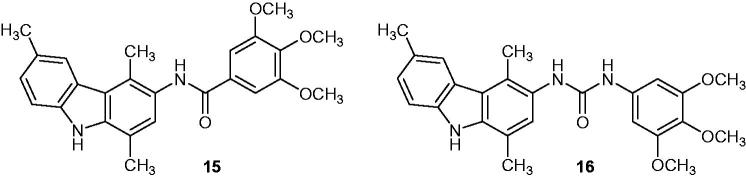
Chemical structures of 1,4-dimethylcarbazole derivatives **15** and **16**.

Two 1,4-dimethylcarbazole derivatives (Figure 22), trimethoxybenzamide **15** and trimethoxyphenylurea **16**, were active against both HL60 (acute promyelocytic leukaemia) and KB (a subline of HeLa) cell lines. These compounds are potent cell proliferation inhibitors, especially **15** that showed the best activities with IC_50_ values of 5.3 µM against HL60 cells and 6.7 µM against KB cells. The anti-proliferative activity was correlated with the inhibition of tubulin polymerization, which ranged from 20 to 50% inhibition^51^.

### 2.19. Guanidinocarbazoles

**Figure 23.**
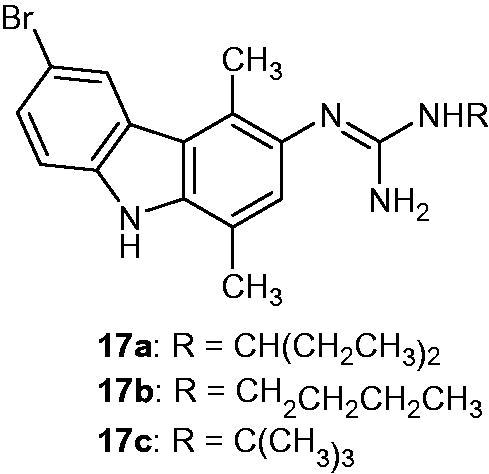
Chemical structures of guanidinocarbazole compounds **17a–c**.

Several alkylguanidines derived from carbazole were prepared and tested for their anti-cancer activity. Three compounds **17a-c** (Figure 23) were tested at 10^−5 ^M against KB and HeLa cell lines, and the best IC_50_ values (3.1, 3.5 and 4 µM, respectively) against HL60 acute promyelocytic leukaemia cell line. Compound **17a**, which was found to be the most active, also demonstrated a high inhibition at 10^−5 ^M against MCF-7, HCT116, PC3 (prostate cancer) and MRC5 (human fatal lung fibroblast) cell lines. Furthermore, fluorescence measurements were carried out and showed that **17a** had some DNA binding properties^52^, which was described as a cycle-dependent cytotoxic activity.

**Figure 24.**
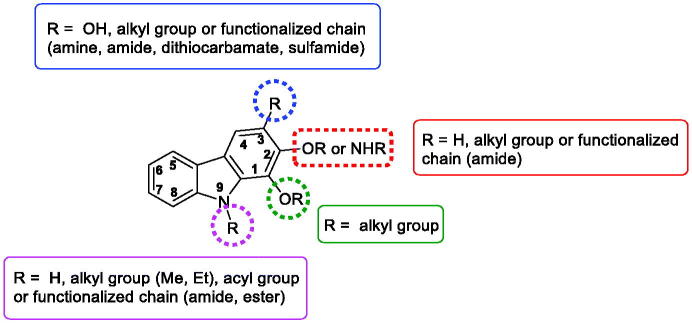
SAR of tricyclic carbazoles.

In summary, various pharmacomodulations in the series of tricyclic carbazoles were performed to obtain new anti-tumour compounds and to investigate the structure–activity relationships. Several substituents were essentially introduced on the nitrogen atom of the indole moiety or on the benzene moiety. Figure 24 shows the substituents that have contributed to the improvement of the anti-tumour activity of the compounds described in this section.

## 3. Tetracyclic fused carbazoles

### 3.1. Tetracyclic carbazoles containing a 5-membered ring

#### 3.1.1. Cyclopenta[c]carbazoles

**Figure 25.**
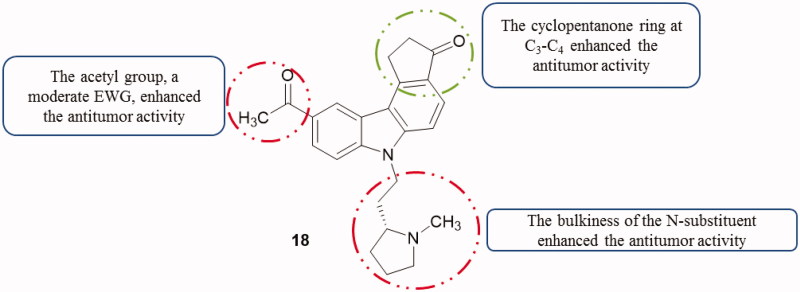
Chemical structure of cyclopenta[*c*]carbazole **18**.

A synthesized series of cyclopenta[*c*]carbazoles were investigated as a p53 activator using two cell lines, HT1080 (lung fibrosarcoma) and RCC45 (renal cell carcinoma) cell lines. The highest activity (EC_50_ value of 0.08 µM) was observed with compound **18** (Figure 25) having an acetyl group at C6 and N9 substituted with (1-methylpyrrolidin-2-yl)ethyl^53^.

#### 3.1.2. Furanocarbazoles

**Figure 26.**
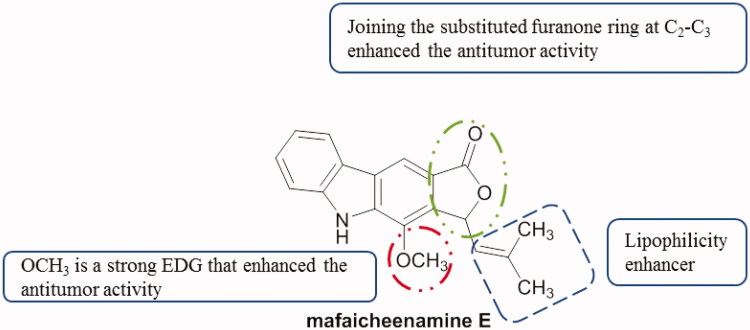
Chemical structure of mafaicheenamine E.

Fourteen compounds, including mafaicheenamines D and E, were isolated from the roots of *Clausena lansium* (Rutaceae) and evaluated against KB, MCF-7 and NCI-H187 cell lines. All compounds were non-cytotoxic against the tested cell lines, except mafaicheenamine E with a methoxy group at C_1_ and bearing a substituted furanone ring on C_2_-C_3_ (Figure 26) which exhibited cytotoxicity against MCF-7 cell line with an IC_50_ value of 10.1 µM^54^.

#### 3.1.3. Pyrrolocarbazoles

**Figure 27.**
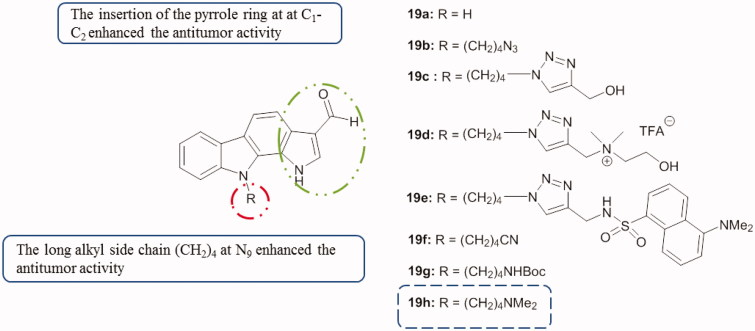
Chemical structures of *N*-10-substituted pyrrolo[2,3-*a*]carbazole derivatives **19a–g**.

Santio et al.^55^ first described the activity of 1,10-dihydropyrrolo[2,3-*a*]carbazole-3-carbaldehyde (DHPCC-9, compound **19a**, Figure 27) as a potent and selective inhibitor for all Pim family members. Novel *N*-10-substituted pyrrolo[2,3-*a*]carbazole-3-carbaldehyde derivatives^56^ were synthesized and evaluated as Pim kinase inhibitors^57^^,^^58^. All cited compounds in Figure 27 showed inhibitory activity of Pim kinases, especially Pim-1 and Pim-3 with IC_50_ comprised between 46 and 490 nM. In vitro anti-proliferative activity of compounds **19f**, **19g** and **19h** was also evaluated using primary human fibroblasts and three human solid cancer cell lines (PA1, PC3 and DU145). These three compounds presented anti-proliferative activities in a micromolar range. Among them, **19h** was the most active compound (0.486 < IC_50_<0.96 µM)^7^.

**Figure 28.**
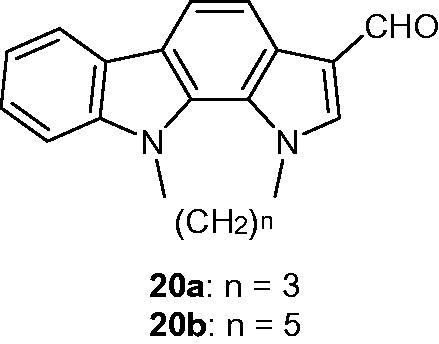
Chemical structures of *N_1_,N_10_*-bridged pyrrolo[2,3-*a*]carbazole-3-carbaldehydes **20a** and **20b**.

Derivatives of N_1_–N_10_-bridged pyrrolo[2,3-*a*]carbazole-3-carbaldehyde^7^ showed interesting inhibitory properties, especially with compounds **20a** and **20b** (Figure 28). Both molecules were presented as Pim-1 and Pim-3 inhibitors (IC_50_ from 0.009 to 0.05 µM). In parallel, compounds **20a** and **20b** with a longer alkyl N_1_–N_10_ bridge exhibited apoptosis-inducing activity toward IPC-81 (acute myeloid leukaemia) cells, but not toward normal fibroblasts^59^.

**Figure 29.**
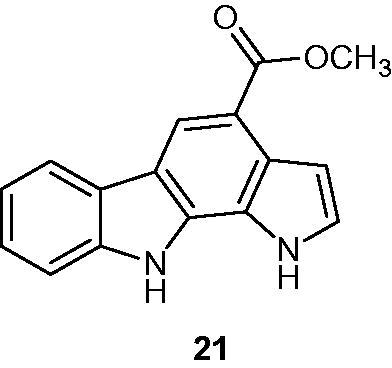
Chemical structure of pyrrolo[2,3-*a*]carbazole **21**.

Other pyrrolo[2,3-*a*]carbazole derivatives with substituent on position 4 were synthesized and their biological activities were evaluated as Pim kinase inhibitors and in vitro anti-proliferative agents. Compound **21** (Figure 29), bearing a methoxycarbonyl group at the 4-position, was found to be active, especially on Pim-3 kinase with IC_50_ around 0.5 µM. The latter also showed anti-proliferative activities on fibroblasts (IC_50_=8 µM) and on PC3 cells (IC_50_ around 6 µM)^60^.

**Figure 30.**
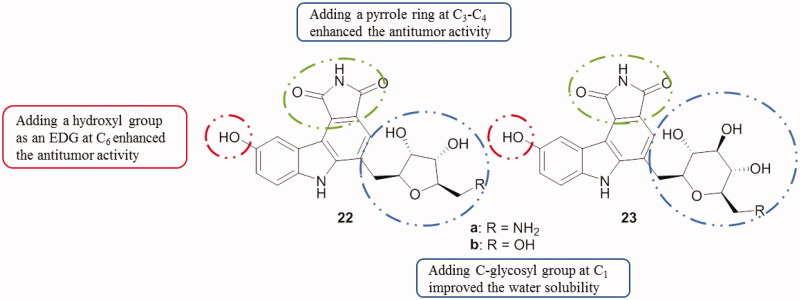
Chemical structures of Chk1 inhibitors **22** and **23**.

Natural C-glycosyl pyrrolo[3,4-*c*]carbazole-1,3(*2H,6H*)-dione derivatives were tested as Checkpoint kinase 1 (Chk1) inhibitors. Compounds **22** and **23** (Figure 30) substituted at the C_1_ with a glycosyl group and at C_6_ with a hydroxyl group were the most active compounds among this series and exhibited IC_50_ values from 0.5 to 9.5 µM^61^.

#### 3.1.4. Pyrazolocarbazoles

**Figure 31.**
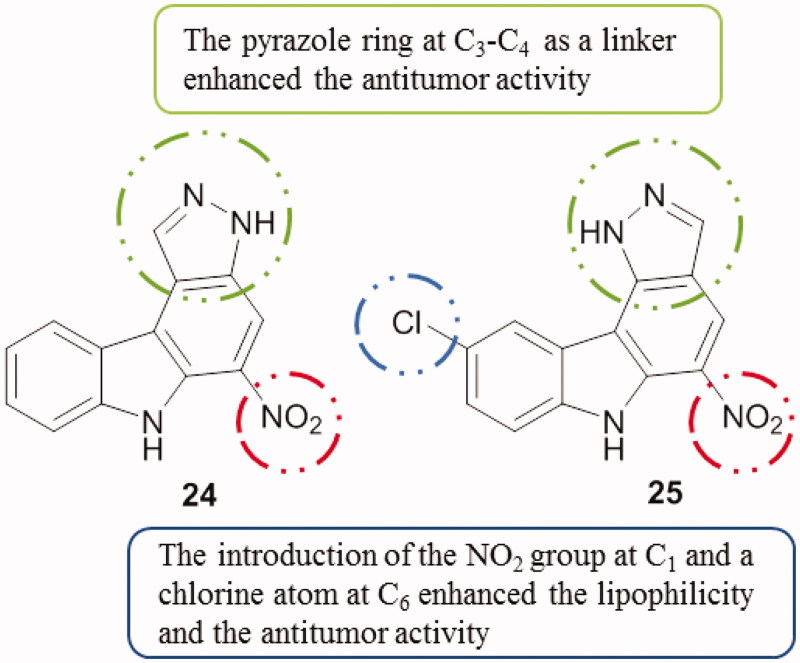
Chemical structures of pyrazolo[3,4-*c*]carbazole **24** and pyrazolo[4,3-*c*]carbazole **25**.

A series of pyrazolocarbazoles [3,4-*c*] and [4,3-*c*] carbazoles was synthesized and tested. Among 3,6-dihydropyrazolo[3,4-*c*]carbazoles, compound **24** (Figure 31) demonstrated to potently inhibit Pim-1 and Pim-3 kinases (IC_50_ from 0.04 to 0.1 µM) and also to be active against proliferative activities of prostatic cancer cells PC3 (IC_50_ was around 3 µM). A 1,6-dihydropyrazolo[4,3-*c*]carbazole, compound **25** (Figure 31), showed the best Pim kinase inhibitory potency toward Pim-3 (IC_50_ around 0.09 µM)^62^.

#### 3.1.5. Isoxazolocarbazoles

**Figure 32.**
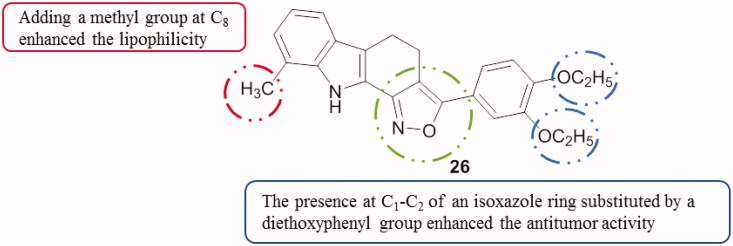
Chemical structure of the isoxazolocarbazole derivative **26**.

A series of carbazole analogues comprising pyrido, isoxazolo, pyrimido and pyrazolo templates were synthesized and evaluated for their cytotoxicity against AGS (gastric cancer) and HeLa cell lines. Among the tested derivatives, 3–(3′,4′-diethoxyphenyl)-9-methyl-4,5-dihydro-10*H*-isoxazolo[3,4-*a*]carbazole (compound **26**, Figure 32) stood out with an IC_50_ value of 0.37 µM against HeLa cells, which was 11 times fold better than the standard ellipticine. All the compounds exhibited a strong in vitro and selective cytotoxicity against HeLa and moderate activities against AGS cell line^63^ (for compound **26**, IC_50_ of 15.12 µM).

**Figure 33.**
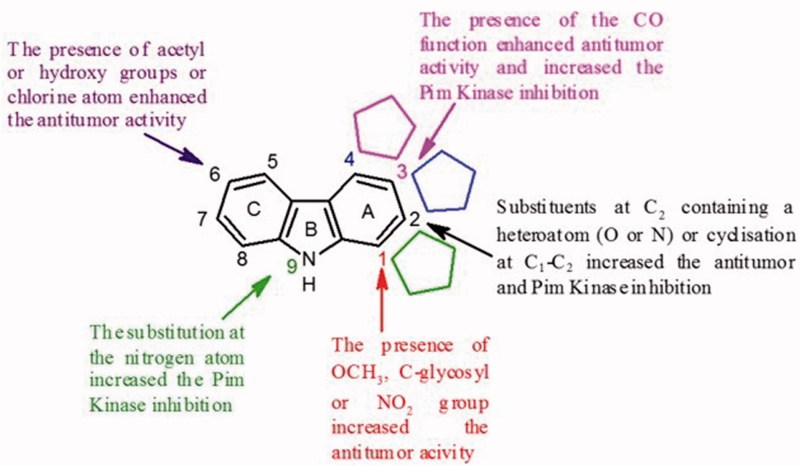
SAR study of tetracyclic carbazoles containing a 5-membered ring.

Briefly, when comparing the mentioned tetracyclic carbazoles containing a five-membered ring, SAR study can be correlated with substitutions at the carbazole ring (Figure 33).

### 3.2. Tetracyclic carbazoles containing a six-membered ring

#### 3.2.1. Benzocarbazoles

**Figure 34.**
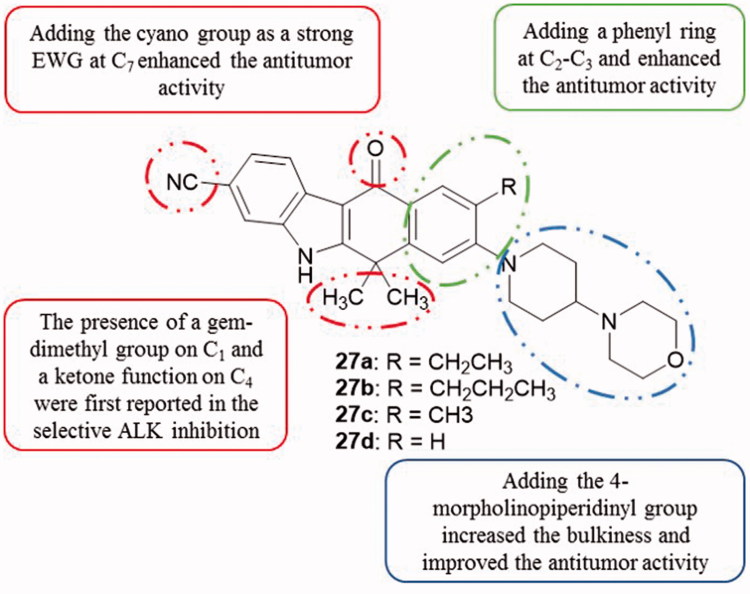
Chemical structures of compounds **27a–d** as ALK inhibitors.

Alectinib/CH5424802 (compound **27a**, Figures 2 and 34) is the second generation ALK inhibitor bearing a *5H*-benzo[*b*]carbazol-11(6*H*)-one structural scaffold which presented a high selective ALK inhibition at a nanomolar scale (IC_50_ value of 1.9 nM)^64^. Additionally, it is a potent anti-proliferative compound against KARPAS-299 cell line (human T cell lymphoma) carrying the nucleophosmin (*NPM)-ALK* fusion gene with an IC_50_ value of 3.0 nM. In vivo studies in mice, using *ALK* fusion gene-positive NSCLC xenograft model, showed that orally administrated compound **27a** significantly regressed tumours. Currently, this compound is being evaluated in phase I/II clinical trials for the treatment of ALK-positive NSCLC^65^.

**Figure 35.**
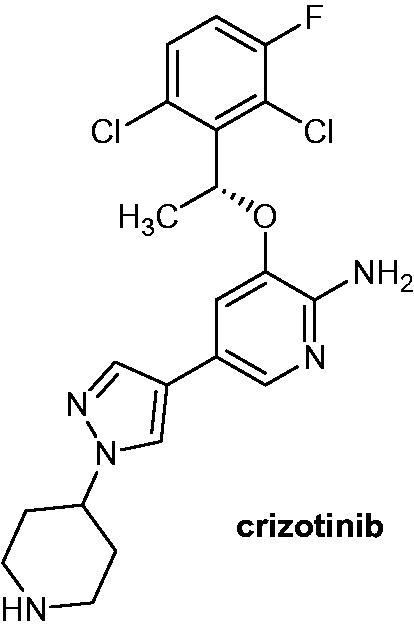
Chemical structures of crizotinib (Xalkori^®^), a second generation of ALK inhibitors.

Compared to the first generation non-carbazole derivative crizotinib (Figure 35), in vitro and in vivo studies showed that alectinib was more potent and selective against wild and mutant ALK. Kodama et al.^66^ showed that the inoculation of alectinib reduced the tumour size and avoided its regrowth. As previously mentioned, alectinib received in 2015 approvals by FDA and EMA for anti-cancer use.

**Figure 36.**
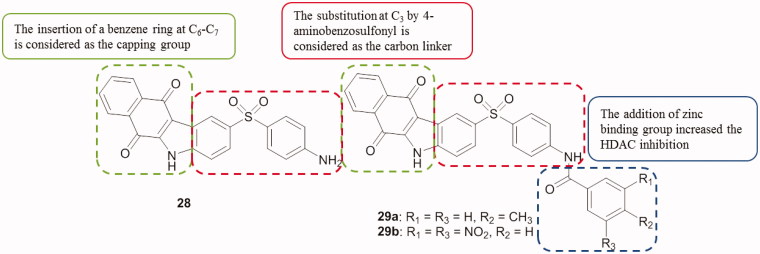
Chemical structures of 2–(4-amino-benzosulfonyl)-5*H*-benzo[*b*]carbazole-6,11-diones **28** and **29a,b**.

A series of 2-(4-aminobenzosulfonyl)-5*H*-benzo[*b*]carbazole-6,11-dione derivatives has been synthesized. In vitro anti-proliferative activity was performed against SiHa (cervical carcinoma) cell lines. Compounds **28**, **29a,b** (Figure 36) exhibited a good cytotoxicity with IC_50_ values of 52.2, 53.8 and 33.5 µM, respectively. The interaction between all compounds and HDAC8 was also carried out by performing molecular docking studies with the use of the GLIDE program^67^.

#### 3.2.2. Pyranocarbazoles

**Figure 37.**
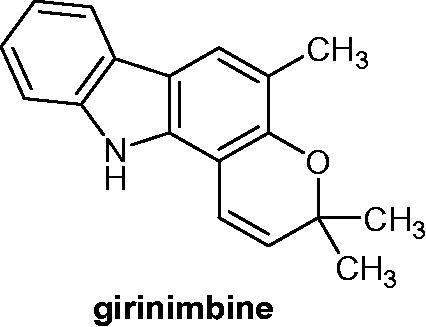
Chemical structure of girinimbine.

Girinimbine (Figure 37), a carbazole alkaloid isolated from the stem bark of *M. koenigii,* had a strong anti-tumour promoting activity. The expression of the Epstein–Barr Virus Early Antigen (EA-EBV) in Raji cells was inhibited by more than 90% when tested at 16 µg/mL (50% inhibition at 22.8 µM). The compound did not alter Raji cell’s viability due to very low cytotoxicity. Girinimbine showed strong anti-oxidant properties comparable to α-tocopherol (vitamin E) and inhibited the superoxide generation in the 12-O-tetradecanoylphorbol-13-acetate (TPA)-induced differentiated HL-60 cells^68^.

**Figure 38.**
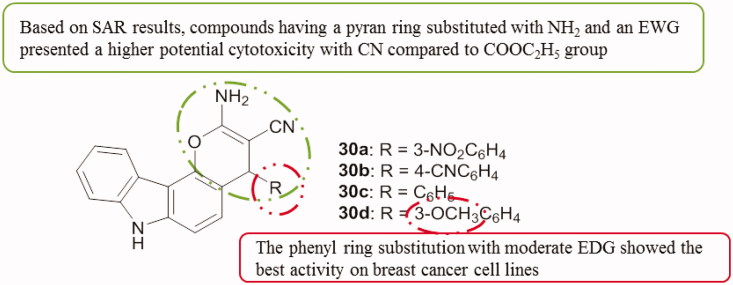
Chemical structures of some pyrano[3,2-*c*]carbazole derivatives **30a–d**.

A series of pyrano[3,2-*c*]carbazole derivatives **30a–d** (Figure 38) showed interesting anti-proliferative activity on different cancer cell lines such as MDA-MB-231, K562, A549 and HeLa with IC_50_ values ranging from 0.43 to 8.05 µM. The MTT cell proliferation and tubulin polymerization assays demonstrated that these compounds induced a G2/M arrest of the cell cycle by inhibiting tubulin and disrupting the microtubule network. The caspase-3 assay demonstrated that the cell death occurred by apoptosis^69^.

**Figure 39.**
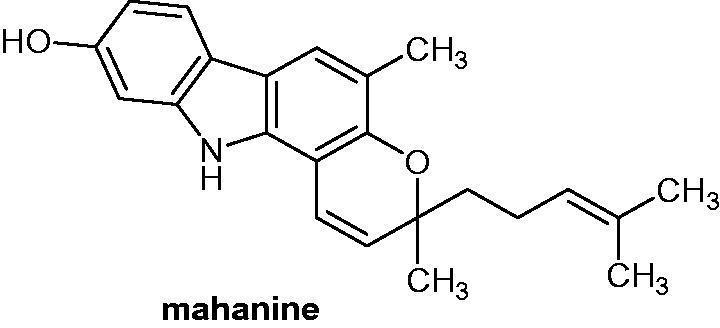
Chemical structure of mahanine.

Mahanine (3,5-dimethyl-3–(4-methylpent-3-en-1-yl)-3,11-dihydropyrano[3,2-*a*]carbazol-9-ol, Figure 39) has been tested in several studies as a single compound or in combination for anti-cancer therapy, for example HCT116 (IC_50_=25.5 µM), HeLa (IC_50_=24.3 µM) and AGS (IC_50_=33.8 µM) cancer cell lines were employed. It showed a growth inhibitory effect with IC_50_ in the micromolar range (from 12.6 to 33.8 µM). Mahanine is also a DNA intercalative cytotoxic molecule, which presented anti-oxidant capacities. On human cancer cell lines, it indicated excellent radical scavenging of the 2,2-diphenyl-1-picrylhydrazyl radical (DPPH•, 9.2 µM), 2,2′-azino-bis(3-ethylbenzothiazoline-6-sulphonic acid radical (ABTS•+, 6837.5 µmol Trolox/g), OH• (12.0 µM) and nitric oxide radical (NO•, 7.8 µM)]. Mahanine showed as well alpha-glucosidase inhibitory activity with IC_50_ value of 21.4 µM^70^.

Das et al.^71^ reported that the combination of mahanine with 5-fluorouracilenhanced the reactive oxygen species (ROS) production, increased the activation of tumour suppressor proteins and suppressed chemo-migration. In another study^72^, the same authors reported that when associated to cisplatin, mahanine could overcome cisplatin-toxicity and drug resistance. Mahanine synergically improved the apoptosis induced by cisplatin in cervical cancer cells and inhibited migration property. The combination at molar ratio 1:4 of cisplatin: mahanine showed a growth inhibitory effect on HeLa and SiHA cell lines (IC_50_ of 1.6–1.8 µM, respectively). This effect on cell inhibition was 10 times higher to the inhibition induced only by mahanine.

#### 3.2.3. Pyridocarbazoles

The commercial analogue of ellipticine, Celiptium^®^ (Figure 1), is active against metastatic breast cancer and acts as an inhibitor of type II topoisomerase. Several studies have also reported apoptosis induction by ellipticine involving the p53 tumour suppressor protein. Prudent et al.^73^ have recently reported a novel mechanism of action of ellipticine as a new inhibitor of the casein kinase CK2 and new analogues are currently developed.

**Figure 40.**
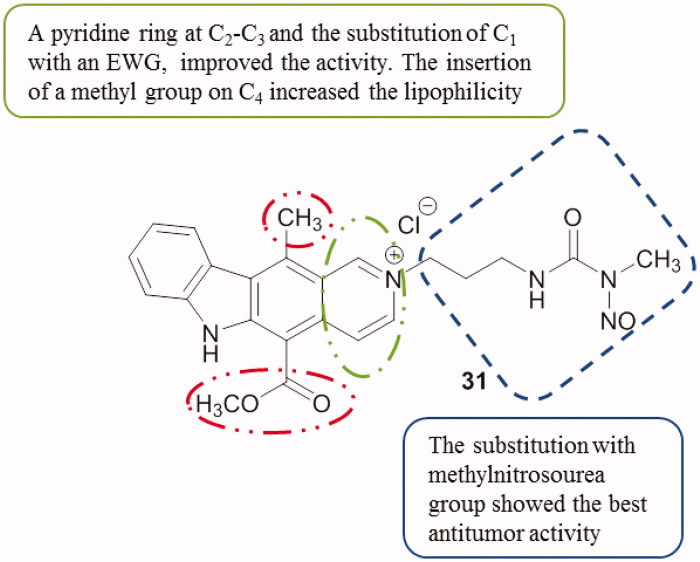
Chemical structure of the pyridocarbazole **31**.

Mori et al.^74^ reported the synthesis of new ellipticine and pyridocarbazole derivatives and their evaluation against HeLa S-3 cell lines. Most of the compounds showed anti-tumour activity with IC_50_ values between 2.50 µM and 60 µM. It appeared that compound **31** (Figure 40), an ellipticinium-analogue linked to a methylnitrosourea group, showed the best anti-tumour activity with an IC_50_ value of 1.3 µM which was two times more potent than ellipticine (2.1 µM).

**Figure 41.**
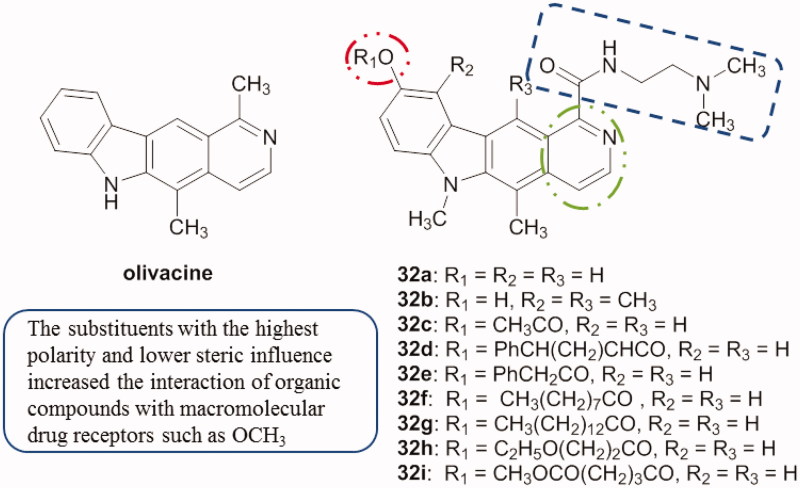
Chemical structures of olivacine and derivatives **32a–i** used for 2 D QSAR analysis.

Quantitative structure activity analysis (QSAA) was carried out on olivacine and compounds **32a–i** (Figure 41) using the software TSAR to determine the structural features responsible for their activity^75^.

**Figure 42.**
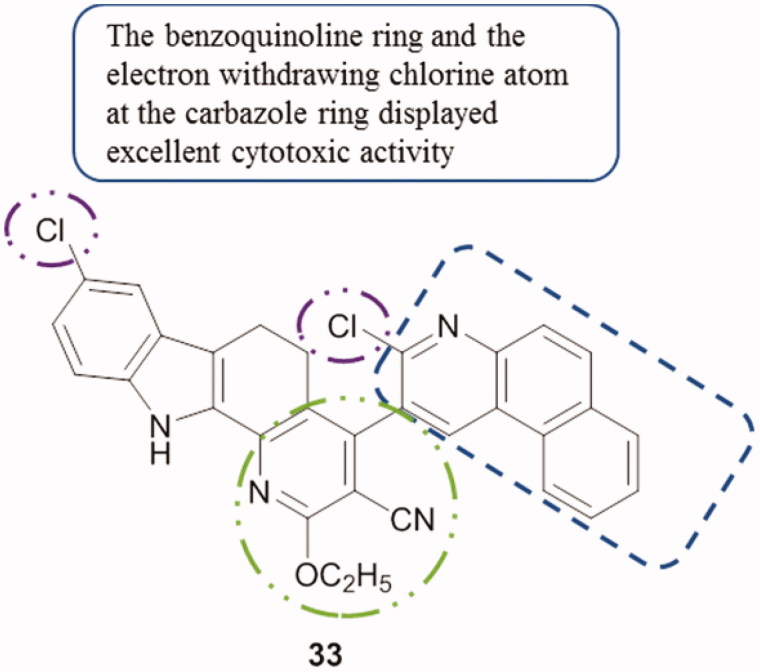
Chemical structure of hetero annulated carbazole compound **33**.

Hetero annulated carbazoles were designed, synthesized and their in vitro cytotoxicity was evaluated against HeLa and MCF-7 cell lines by MTT assay and compared to the standard drug ellipticine. Compound **33** (Figure 42) demonstrated 1.2-fold stronger activity than ellipticine’s cytotoxic activity against HeLa. Then, molecular docking studies were carried out using CK2 as a target, in which compound **33** showed the lowest binding energy and best ligand efficiency^76^. SAR studies revealed that, the compound bearing the pyrimido moiety and the electron-withdrawing chlorine in the carbazole displayed excellent cytotoxic activity (IC_50_ value of 8.11 µM) against HeLa cells.

**Figure 43.**
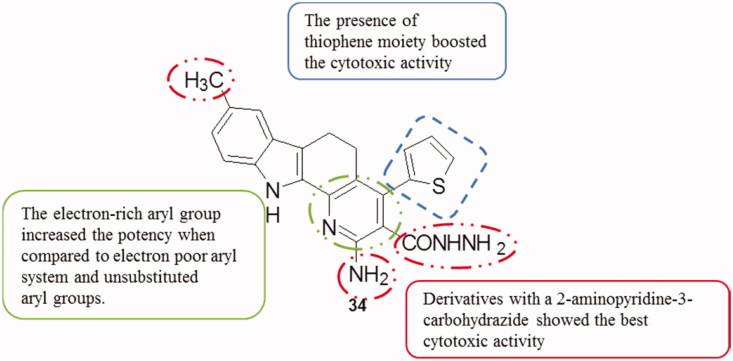
Chemical structure of the pyridocarbazole **34**.

The in vitro cytotoxicity of the pyrido[2,3-*a*]carbazoles was evaluated by SRB (sulfo-rhodamine B) assay against MCF-7, HeLa and A549 cell lines. Among these derivatives, compound **34** (Figure 43) showed the best activity with an IC_50_ of 13.42 µM against HeLa cells (cisplatin, 13.20 µM). All the designed compounds demonstrated a higher potency against HeLa than against the other tested cell lines^77^.

**Figure 44.**
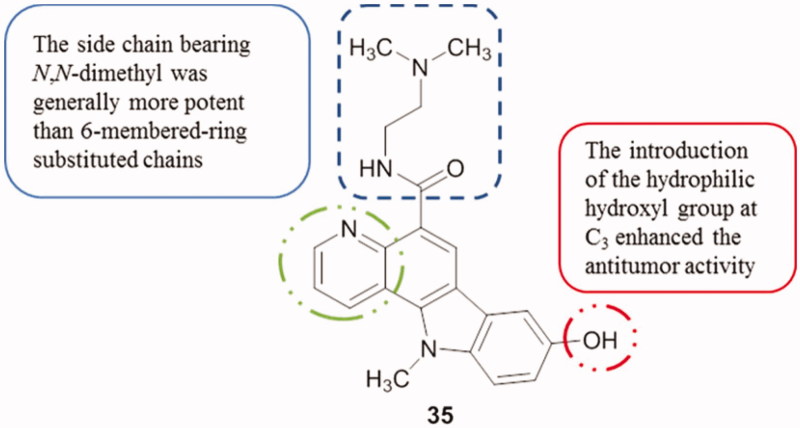
Chemical structure of pyridocarbazole **35**.

Pyrido[3,2-*α*]carbazole derivatives and their analogues were tested against A549 and HT29 cell lines with IC_50_ values ranging from 0.07 µM to 4.45 µM. For example, compound **35** (Figure 44) was active against A549 cells with an IC_50_ value of 0.07 µM and with 0.11 µM against HT29 cells^78^.

**Figure 45.**
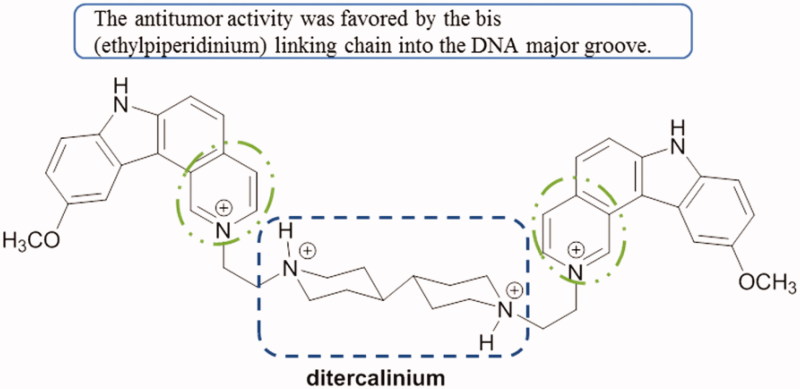
Chemical structure of ditercalinium.

Ditercalinium is a dimer of two 7*H*-pyrido[4,3-*c*]carbazole units (Figure 45). It is a bis-interacting agent in the major groove of DNA. NMR studies and X-ray crystal structure revealed that both rings of the dimer allowed intercalation with base pairs and caused structural changes in the DNA^79^.

#### 3.2.4. Pyrimidocarbazoles

**Figure 46.**
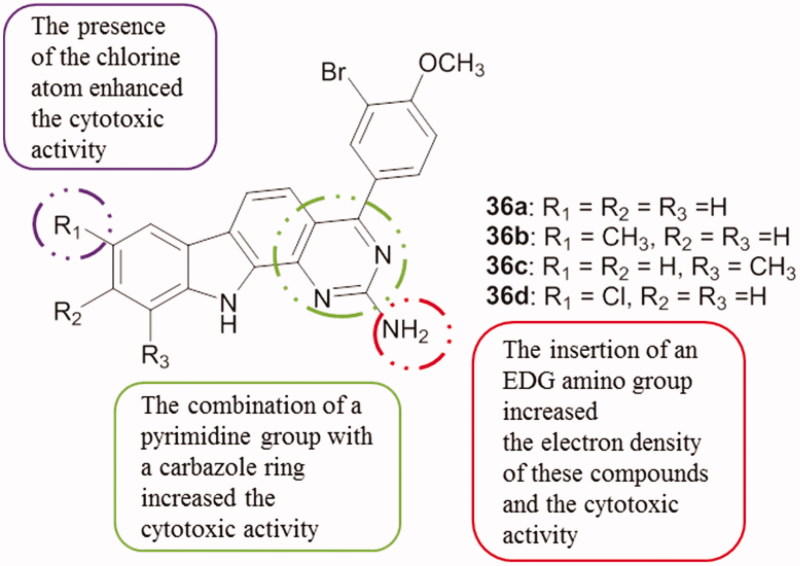
Chemical structures of pyrimido[4,5-*a*]carbazole derivatives **36a–d**.

Among pyrimidocarbazole derivatives **36a–d** (Figure 46), 2-amino-4-(3′-bromo-4′-methoxyphenyl)-8-chloro-11*H*-pyrimido[4,5-*a*]carbazole **36d** showed the best cytotoxic efficacy against both MCF-7 and A-459 cancer cell lines (IC_50_ value of 20 and 25 µM, respectively). Compounds **36a–c** also exhibited stronger cytotoxic activity against MCF-7 cell lines^80^.

#### 3.2.5. Oxazinocarbazoles

**Figure 47.**
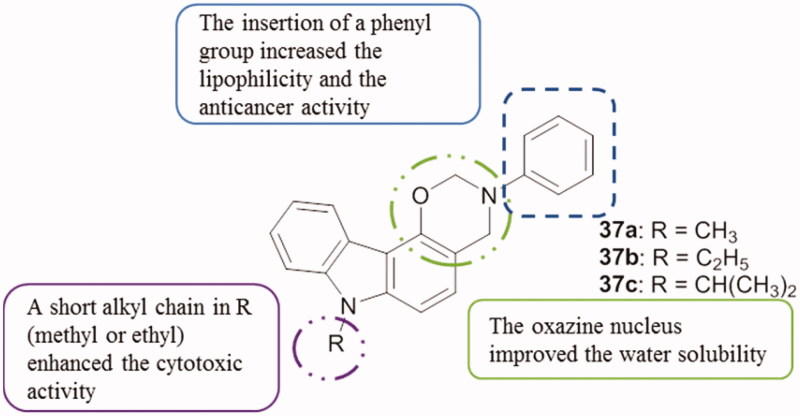
Chemical structures of oxazinocarbazole derivatives **37a–c**.

A set of various oxazinocarbazoles was synthesized^81^ and their activities were studied using a CE-based assay for CK2 activity measurement, a cytotoxicity assay using IPC-81 cells. Three oxazinocarbazoles **37a–c** (Figure 47) showed CK2 inhibition with IC_50_ values of 8.7, 14.0 and 1.40 µM, respectively. Another test using again the IPC-81 cells was then performed, and those compounds demonstrated the ability to induce leukaemia cell death with IC_50_ values between 57 and 62 µM^81^.

#### 3.2.6. Miscellaneous

**Figure 48.**
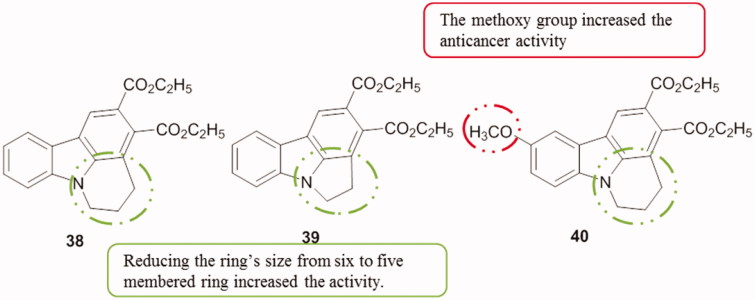
Chemical structures of the tetracyclic carbazoles **38**, **39**, and **40**.

The cytotoxicity of three dihydro-4*H*-pyrido[3,2,1*-jk*]carbazole derivatives was evaluated against HeLa cell lines. Carbazoles **38**, **39** and **40** (Figure 48) showed moderate activity with IC_50_ values of 19.80, 17.46 and 18.76 µM, respectively^82^.

**Figure 49.**
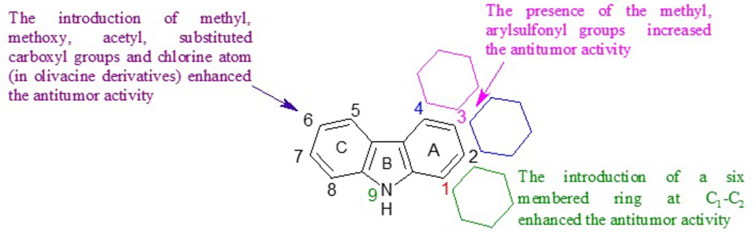
SAR of tetracyclic carbazoles containing a 6-membered ring.

Briefly, when comparing the mentioned tetracyclic carbazoles containing a six-membered ring SAR can be correlated with substitutions at the carbazole ring (Figure 49).

### 3.3. Tetracyclic carbazoles containing a 7-membered ring

#### 3.3.1. 1,4-Thiazepan-3-ones fused carbazoles

**Figure 50.**
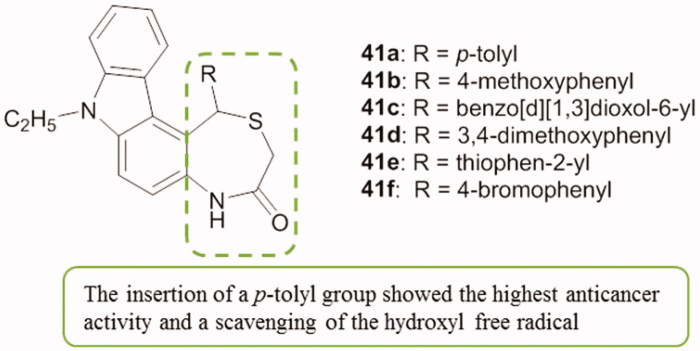
Chemical structures of the 1,4-thiazepan-3-ones fused with carbazoles **41a–f**.

Several 1,4-thiazepine derivatives fused with carbazole skeleton underwent in vitro cytotoxic studies. Compounds **41a–f** (Figure 50) showed selective cytotoxicity towards HCT 116 cell lines with inhibition rates of 51.57–62% at 1 mg/mL^83^.

## 4. Pentacyclic fused carbazoles

### 4.1. Pentacyclic fused carbazoles containing two 5-membered rings

**Figure 51.**
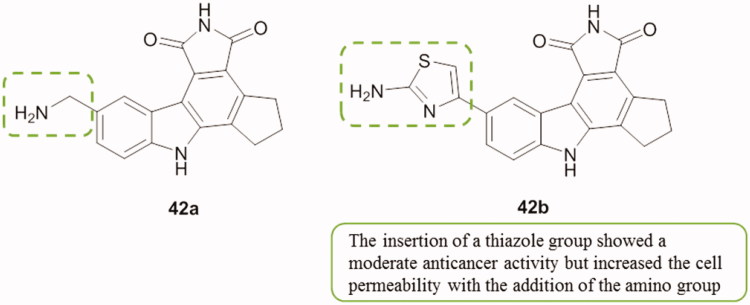
Chemical structures of 3-substituted-pyrrolocarbazole analogs **42a** and **42b**.

A series of 3-substituted-pyrrolocarbazole analogues (3-aroyl-derived pyrrolocarbazoles) was synthesized and evaluated as PARP-1 inhibitors. They were also tested in a cell-based assay that evaluated their ability to attenuate the depletion of NAD^+^ levels following hydrogen peroxide insult in PC12 (rat pheochromocytoma) cells. Results showed that two analogues, compounds **42a** and **42b** (Figure 51), displayed potent enzyme activity with IC_50_ values of 18 and 25 nM, respectively, as well as high cell permeability (100% NAD^+^ recovery at 30 µM)^84^.

### 4.2. Pentacyclic fused carbazoles containing a five-membered ring and a six-membered ring

#### 4.2.1. Tetrahydroindolo[2,3-b]carbazoles

**Figure 52.**
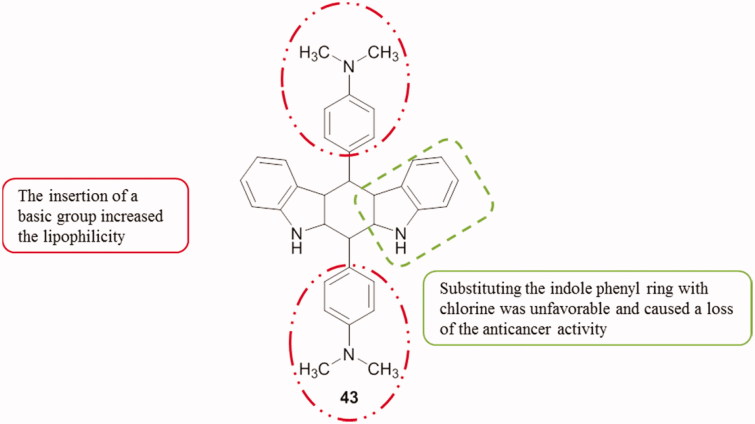
Chemical structure of tetrahydroindolocarbazole **43**.

Tetrahydroindolo[2,3-*b*]carbazoles were synthesized to undergo a one dose screening at 10^−5 ^M, followed by a five dose screening for the best compounds using the NCI 60 cell lines list. Compound **43** (Figure 52) exhibited the highest anti-cancer activity with growth inhibition at lowest mean value of 21.63%, and GI_50_ values ranging from 1.07 to 9.56 µM against the tested cell lines^85^.

**Figure 53.**
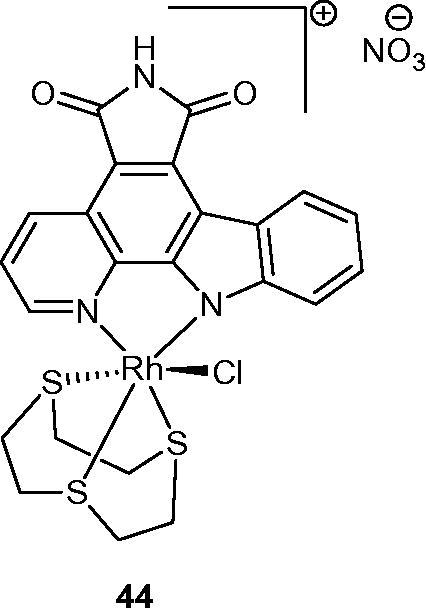
Chemical structure of pyridocarbazole-rhodium complex **44**.

Several inert metal complexes such as pyridocarbazole-rhodium (III) were synthesized and characterized by X-ray crystallography. Stability studies were carried out including evaluation of Pim-1 kinase inhibitory activity. Compound **44** (Figure 53) was found to be a stable rhodium (III) complex and extremely potent inhibitor of Pim-1 kinase (IC_50_ around 160 pM)^86^.

**Figure 54.**
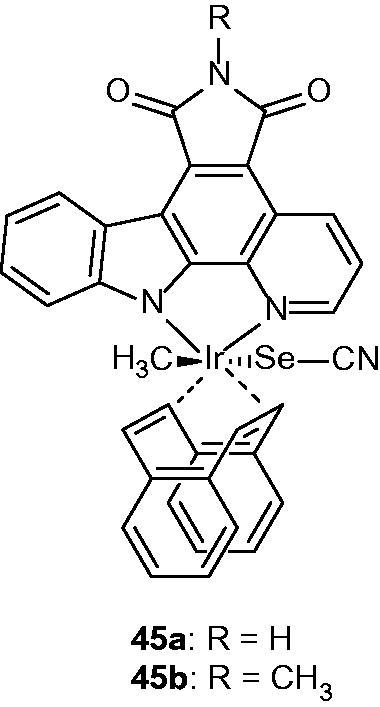
Chemical structures of iridium complex **45a** and its N-methylated derivative **45b**.

The iridium–pyridocarbazole complexes **45a** and **45 b** (Figure 54) are highly photocytotoxic compounds. Their anti-angiogenic properties were investigated in a 3D angiogenesis assay. It resulted that **45a** and **45b** are light-independent potent anti-angiogenic agents, very active on the vascular endothelial growth factor^87^.

### 4.3. Pentacyclic carbazoles containing two six-membered rings

#### 4.3.1. 7H-Dibenzo[c,g]carbazole

**Figure 55.**
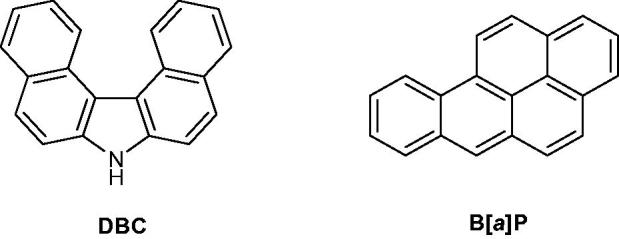
Chemical structure of DBC and B[*a*]P.

7*H*-Dibenzo[*c,g*]carbazole (DBC), benzo[*a*]pyrene (B[*a*]P) (Figure 55) and several binary mixtures of both compounds were assessed. The biological activity of the binary mixtures was investigated in the HepG2 and WB-F344 (liver cancer) cell lines and the Chinese hamster V79 cell line. These compounds showed an important biological activity on human carcinogens acting on a micro cellular level by modifying cytochrome CYP1A1 expression^88^.

#### 4.3.2. Murrayazolinine

**Figure 56.**
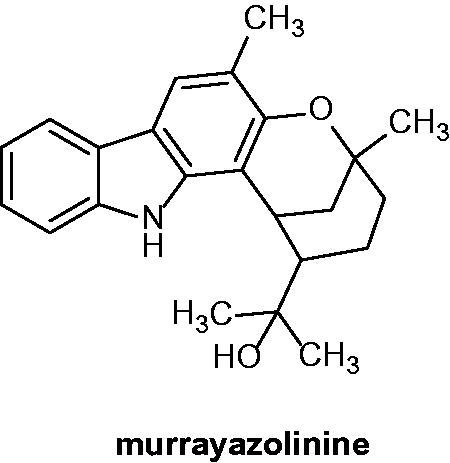
Chemical structure of murrayazolinine.

Murrayazolinine (Figure 56) was isolated from *M. euchrestifolia* (Rutaceae) and was evaluated against HL-60 cell line. Murrayazolinine displayed a significant interaction with the caspase-9/caspase-3 pathway, leading to the cellular apoptosis^40^.

#### 4.3.3. Carbazole–amonafide structural hybrids

**Figure 57.**
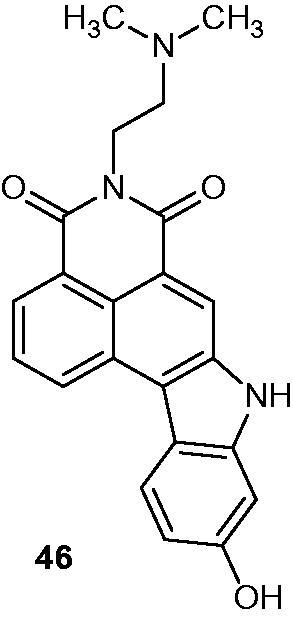
Chemical structure of carbazole-amonafide structural hybrid lead candidate **46**.

Preliminary anti-proliferative assays revealed that structural hybrids [4,5-*bc*]carbazole-amonafide derivatives possessed a good cytotoxic activity with IC_50_ values in the sub-micromolar to micromolar range against HTC116 cell line, and were also selective for cancer cells when compared to a HEK293 (non-cancerous human embryonic kidney) cell line. Compound **46** (Figure 57) was the lead candidate with an IC_50_ value of 0.8 µM against HTC116 cell line and an IC_50_ value above 40 µM against normal cells^89^.

## 5. Hexacyclic fused carbazoles

### 5.1. Indenopyrrolocarbazoles

**Figure 58.**
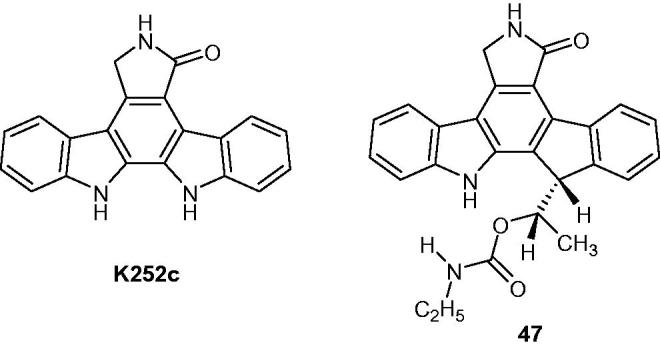
Chemical structures of K252c the indenopyrrolocarbazole, compound **47**.

A strong lead candidate, compound **47** (Figure 58), was synthesized from staurosporine aglycone (K252c)^90^. The structure–activity relationship showed that compound **47** is a powerful tropomyosine kinase TrkA inhibitor; therefore, it was selected as a proof of concept for in vitro and in vivo studies^53^.

### 5.2. Indazolopyrrolocarbazoles

**Figure 59.**
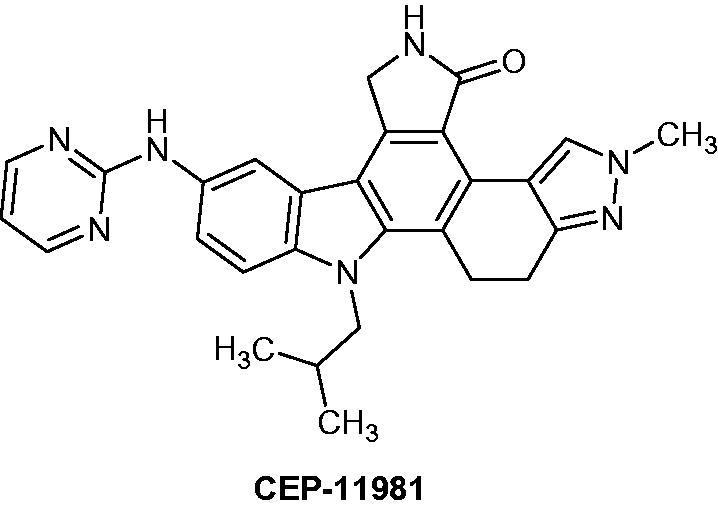
Chemical structure of CEP-11981.

11–(2-Methylpropyl)-12,13-dihydro-2-methyl-8-(pyrimidin-2-ylamino)-4*H*-indazolo[5,4-*a*]pyrrolo[3,4-*c*]carbazol-4-one (CEP-11981) (Figure 59) is a potent orally active inhibitor of multiple tyrosine kinase receptors (e.g. tyrosine kinase with immunoglobulin and EGF homology domains (TIE2), vascular endothelial growth factor receptor (VEGFR 1–3) and fibroblast growth factor receptor, FGFR1), which are potent targets for tumour angiogenesis and vascular maintenance. Furthermore, CEP-11981 exhibits excellent permeability, metabolic stability and pharmacokinetic properties. It was advanced into full development and was in clinical phase I study^91^.

### 5.3. Indolopyrrolocarbazoles

**Figure 60.**
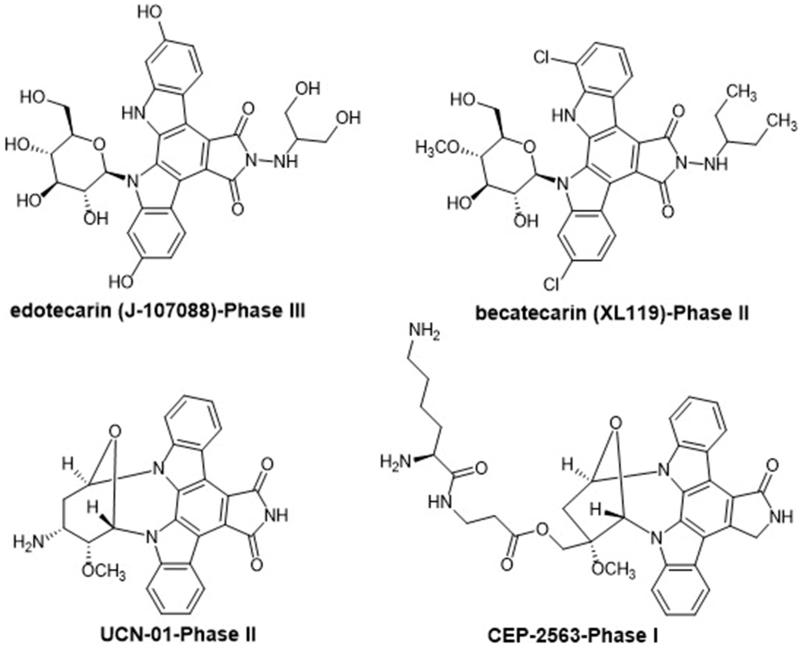
Chemical structures of the synthetic indolocarbazoles in clinical trials.

Among the carbazole derivatives, four synthetic indolopyrrolocarbazoles (Figure 60) are currently in clinical trials for cancer therapy. CEP-2563 is active against MTC (medullary thyroid carcinoma) and blocked tyrosine kinase receptors such as Trk family and the platelet-derived growth factor (PDGF) receptor tyrosine kinase^92^. Edotecarin (J-107088) and becatecarin (XL119) which both could intercalate into DNA and edoteacrin could additionally stabilize the DNA–topoisomerase I complex. Edotecarin (J-107088) is currently in phase III trials (Pfizer) and becatecarin (XL119) (NCI) is in clinical trials (phase II) and represent promising approaches for the cancer therapy. UCN-01 is a protein kinase C (PKC) inhibitor and is currently in Phase II trials (NCI) for its activity against pancreatic, lymphoma and breast cancers^92^.

#### 5.3.1. Staurosporine and analogues

**Figure 61.**
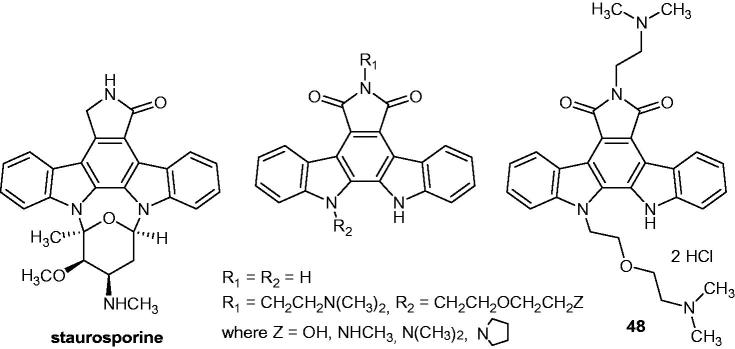
Chemical structures of staurosporine, indolocarbazole analogs and compound **48**.

A series of indolocarbazoles and staurosporine analogues (Figure 61) were synthesized and tested as anti-proliferative agents against HUVEC (Human Umbilical Vein Endothelial Cells), LoVo (colorectal adenocarcinoma), DLD-1 (colorectal adenocarcinoma) and ST-486 (Burkitt's lymphoma) cell lines. Their anti-angiogenesis activity was also investigated by capillary tube formation in 3-D matrigel matrix. Acero et al.^93^ observed on all cell lines that the dimethylaminoalkyl chain in R_1_ (Figure 61) enhances both activity and selectivity. Analog **48** (Figure 61), with an IC_50_ of 0.1 µM against HUVEC, was one of the most active compounds and the most selective one. The in vivo anti-angiogenic assay using the Lewis lung mice carcinoma model revealed that no tumour reduction was observed, although a slight reduction in metastasis number was noticed^93^^,^^94^.

#### 5.3.2. Streptocarbazoles A and B

**Figure 62.**
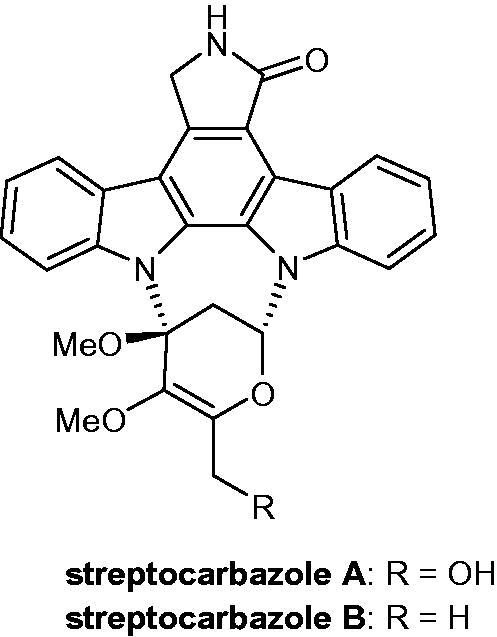
Chemical structures of streptocarbazoles A and B.

Isolated from the marine-derived actinomycetes strain *Streptomyces* sp. FMA, streptocarbazoles A and B (Figure 62) were tested as anti-tumoural agents. Streptocarbazole A was cytotoxic against HL-60, A-549 (lung carcinoma), P388 (leukaemia) and HeLa cell lines, with IC_50_ values of 1.4, 5.0, 18.9 and 34.5 µM, respectively, and could arrest the cell cycle of HeLa cells at the G2/M phase (at a concentration of 10 µM). Streptocarbazole B was only active against P388 and HeLa cells, demonstrating IC_50_ values of 12.8 and 22.5 µM, respectively^95^.

#### 5.3.3. Methylenedioxy- and ethylenedioxy-fused indolopyrrolocarbazoles

**Figure 63.**
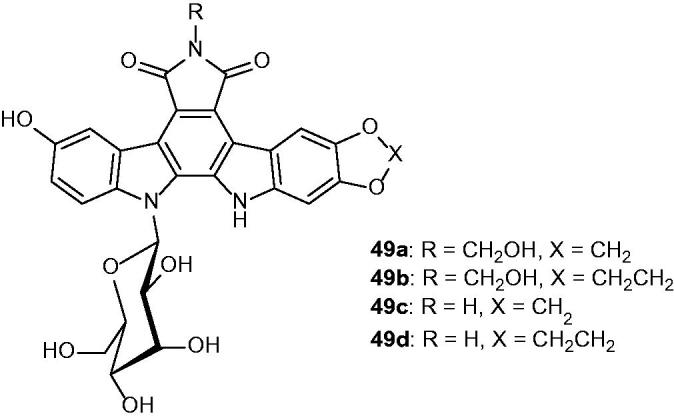
Chemical structures of new indolopyrrolocarbazoles **49a–d**.

The biological activity of indolo[2,3-*a*]carbazole derivatives (Figure 63) was determined as potential anti-cancer agents. Among the analogues, compounds **49a–d** were the most potent compounds against human topoisomerase I and exhibited inhibitory activities with IC_50_ values in the micromolar range (from 3.2 to 5.4 µM)^96^.

**Figure 64.**
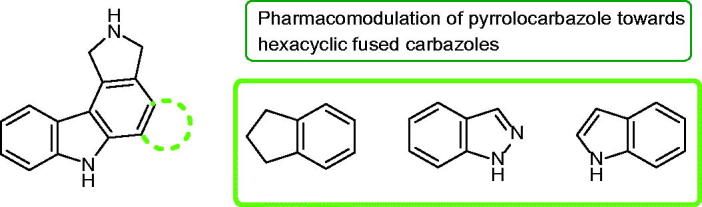
From pyrrolocarbazole, a growing approach to design new hexacyclic fused carbazoles as anti-cancer drugs.

In summary, the scaffold pyrrolocarbazole was extensively used as a part of hexacyclic fused carbazoles. Three sub-series of related compounds were developed, namely indeno-, indazo- and indolo-pyrrolocarbazoles (Figure 64). For some the additional presence of a sugar moiety is also to notice (e.g. edotecarin, compounds **49a–d**). It is an important point to modulate their physicochemical properties such as hydrosolubility and then to facilitate in vivo investigation.

### 5.4. Indolopyrazolocarbazoles

**Figure 65.**
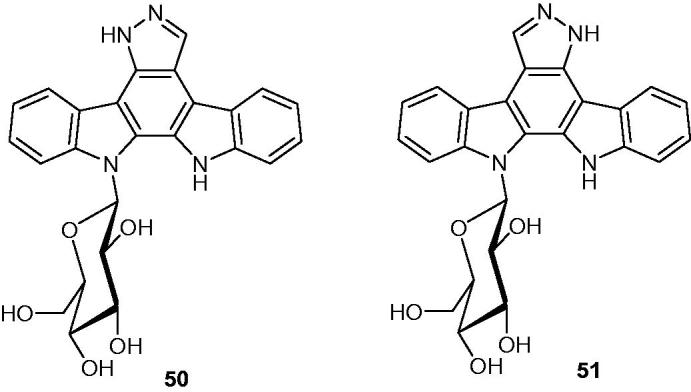
Chemical structures of indolocarbazoles **50** and **51**.

Glycosylated indolopyrazolocarbazole analogues of K252c (Figure 58) have been synthesized to improve their cellular potencies leading to two compounds: 13-(1-deoxy-β-d-glucopyranos-1-yl)-12,13-dihydro-5*H-*indolo[2,3-*a*]pyrazolo[3,4-*c*]carbazole **50** and 12-(1-deoxy-β-d-glucopyranos-1-yl)-12,13-dihydro-*5H*-indolo[2,3-*a*]pyrazolo[3,4-*c*]carbazole **51** (Figure 65)^97^. Interestingly, compounds **50** and **51** were active toward HCT116 (human colon carcinoma) cell line with similar IC_50_ values of 12 and 11 µM, respectively.

### 5.5. Indolopyrimidocarbazoles and related

**Figure 66.**
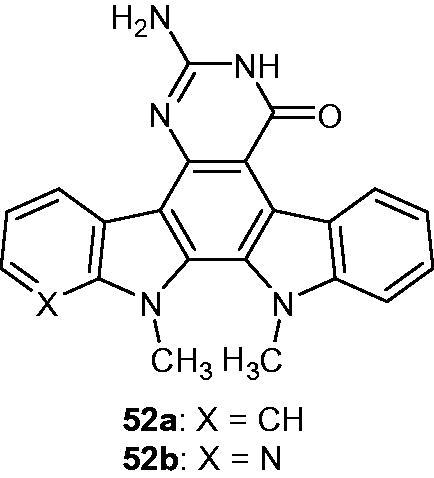
Chemical structures of indolopyrimidocarbazole **52a** and azaindolopyrimidocarbazole **52b**.

A series of indolo[2,3-*a*]pyrimido[5,4-*c*]carbazoles and azaindolopyrimidocarbazoles was synthesized and their anti-cancer activity was evaluated through topoisomerase II inhibition and in cellulo assay using the NCI-60 cell line. Although no topoisomerase II inhibition was observed, compound **52a** (Figure 66) was found to inhibit in vitro the growth of HCT-15 (colon carcinoma), SK-MEL-2 (melanoma), and ACHN, CAKI-1 and UO-31 (renal adenocarcinoma) cell lines with GI_50_ values in the low micromolar range. The less toxic azaindolocarbazole **52b** (Figure 66) also showed cytostatic activity against NCI-H522 (non-small-cell lung cancer) and UO-31 cell lines^98^.

**Figure 67.**
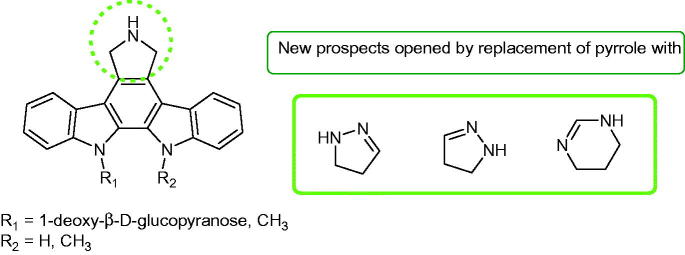
Pharmacomodulation works by replacement of pyrrole moiety.

In the particular point of the replacement of the pyrrolo moiety of indolopyrrolocarbazole either by a pyrazolo or by a pyrimido ring systems (Figure 67), new active hexacyclic derivatives demonstrated cytotoxic activity on colon carcinoma cell lines (e.g. HCT116, HCT-15).

## 6. Heptacyclic fused carbazoles

### 6.1. Carbazole derivatives of ursolic acid

**Figure 68.**
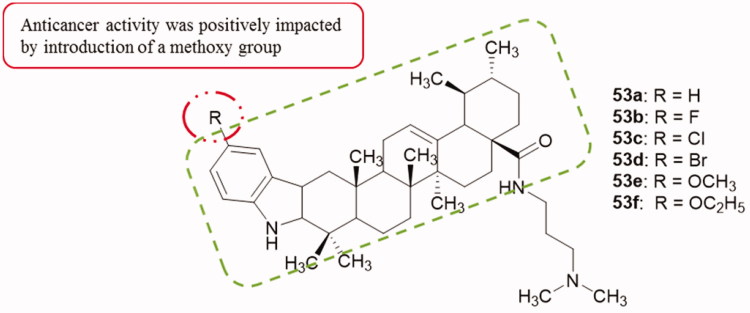
Chemical structures of carbazole derivatives of ursolic acid **53a–f**.

A series of carbazole derivatives of ursolic acid was synthesized and assayed against two human liver cancer cell lines (SMMC-7721 and HepG2) using the MTT colorimetric method. From the results, compounds **53a–f** (Figure 68) displayed pronounced cytotoxic activities with IC_50_ values below 10 µM. Compound **53e** was found to be the most active compound with IC_50_ values of 1.08 ± 0.22 and 1.26 ± 0.17 µM against SMMC-7721 and HepG2 cells, respectively, comparable to those of doxorubicin. In addition, **53e** showed reduced cytotoxicity against noncancerous LO_2_ cells with an IC_50_ value of 5.75 ± 0.48 µM^99^.

## 7. Conclusion

**Figure 69.**
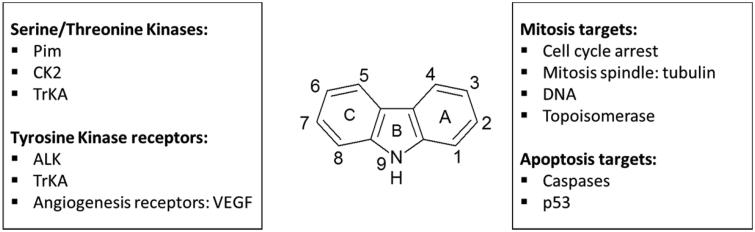
Major anti-tumor activities of carbazole derivatives.

Cancer is a very complex disease and the increase of the biological targets can be synergistically coordinated to relieve patients from cancer burden. Many new cancer therapies have been developed in the last years, but this research field still presents many challenges. Among the natural products, the carbazole alkaloids have shown several biological activities (Figure 69). Since 2012, we presented the major anti-tumoural activities of natural and synthetic carbazole derivatives.

In a recent study, Iman et al.^100^ showed that the combination of many mechanisms of action were observed in the case of girinimbine which resulted in an induction of G0/G1 phase arrest, an upregulation of two cyclin-dependent kinase proteins p21 and p27, an activation of caspase-3 and caspase-9, downregulation of Bcl-2 and upregulation of Bax in girinimbine-treated cells. Another activity was seen on the upregulation of p53. Induction of apoptosis by girinimbine was also investigated in vivo by using zebrafish embryos, with results demonstrating significant distribution of apoptotic cells in embryos after a 24-h treatment period^100^. Some compounds are currently following clinical trial phases and the optimal structure has not yet been found. Carbazole derivatives have been recently described by Diaz et al. for their anti-tumour activity with the microtubule targeting and could inhibit tubulin assembly^101^. Many potential compounds can be the future candidate for the cancer chemotherapy, with the purpose of a multi-target therapy.
